# Risk factors for gastric cancer: an umbrella review of systematic reviews and meta-analyses

**DOI:** 10.3389/fonc.2025.1564575

**Published:** 2025-06-26

**Authors:** Jin Long Liang, Hui Ming Yuan, Chao Quan, Jun Qiang Chen

**Affiliations:** ^1^ Department of Gastrointestinal Surgery, The First Affiliated Hospital of Guangxi Medical University, Nanning, Guangxi, Nanning, China; ^2^ Guangxi key Laboratory of Enhanced Recovery after Surgery for Gastrointestinal Cancer, Nanning, China; ^3^ Guangxi Clinical Research Center for Enhanced Recovery after Surgery, Nanning, China; ^4^ Guangxi Zhuang Autonomous Region Engineering Research Center for Artificial Intelligence Analysis of Multimodal Tumor Images, Nanning, China; ^5^ Department of Surgery, University of Michigan Medical School, Ann Arbor, MI, United States; ^6^ Department of Urology, Xiangya Hospital, Central South University, Changsha, Hunan, China

**Keywords:** gastric cancer, risk factor, umbrella review, meta-analysis, system review

## Abstract

**Background:**

This umbrella review aims to critically appraise and synthesize epidemiological evidence from meta-analyses to identify and classify risk and protective factors associated with gastric cancer.

**Methods:**

PubMed, Embase, Web of Science, and the Cochrane were used to search, including meta-analyses up to April 2024. Emphasis was placed on non-interventional studies, and the inclusion criteria focused on meta-analyses that involved diverse ethnic groups and genders from various countries and settings. Two reviewers independently evaluated the methodological quality using the AMSTAR tool and classified evidence strength based on established criteria.

**Results:**

Of 245 meta-analyses meeting inclusion criteria, 117 unique risk factors were identified, including 77 significantly associated factors (42 adverse and 35 protective) and 40 non-significant factors. 17 (14.5%) risk factors were classified as class I or II evidence in this umbrella review. Protective factors included cruciferous vegetable intake, total cholesterol (TC), HDL cholesterol (HDL-C), NSAIDs, β-carotene, vitamins, and dietary polyphenols. Risk factors included depression, *Helicobacter pylori (Hp)* infection, dermatomyositis, and Graves’ disease. Class III evidence confirmed that aspirin, non-aspirin NSAIDs, soy food intake, non-fermented soy food intake, physical activity, vitamin A, ginseng, dietary fiber, tooth brushing frequency, folate, and green tea consumption were associated with reduced GC risk. Conversely, Epstein-Barr virus infection, red meat, processed meat, intestinal metaplasia, gastric atrophy, a western-style diet, dietary cholesterol, dietary salt, and proton pump inhibitors were linked to higher GC risk.

**Conclusion:**

This umbrella review identified 77 risk factors significantly associated with gastric cancer (GC), the majority of which are linked to personal traits and lifestyle behaviors. These findings enhance our understanding of GC etiology and can inform strategies to reduce incidence, delay progression, and alleviate the global burden.

**Systematic review registration:**

https://www.crd.york.ac.uk/PROSPERO/, identifier CRD42023447199.

## Introduction

Gastric cancer (GC) is a prevalent digestive system malignancy with a poor prognosis. Ranking fifth in incidence among common cancers worldwide, its distribution varies considerably across geographic regions and ethnic groups (1, 2). Early-stage GC is often asymptomatic or presents with nonspecific symptoms, resulting in delayed diagnosis and treatment. Consequently, many cases are diagnosed at advanced stages, substantially worsening patient outcomes (3). Data show that the 5-year survival rate for early GC exceeds 90%, but it drops significantly to below 20% once the disease progresses to intermediate or late stages (4, 5). GC is a multifactorial disease influenced by both modifiable and non-modifiable risk factors. While genetic predispositions and aging are beyond control, environmental exposures, infections, dietary habits, drug use, and psychological factors represent modifiable contributors, highlighting the potential for prevention through targeted interventions. Numerous meta-analyses have identified various risk factors for GC (6–18). Among these, environmental factors are increasingly recognized as significant. Excessive smoking, alcohol consumption, high-salt and fried food intake, and the consumption of red and processed meats are strongly associated with an elevated GC risk in a dose- or time-dependent manner (1, 19, 20). Insufficient fruit intake, physical inactivity, and obesity also increase the risk of GC (21–23). Environmental pollutants, including poor drinking water quality, contaminated water sources, and soil pollution, have also been linked to higher GC incidence. *Helicobacter pylori (Hp)* infection remains a primary high-risk factor, with early eradication showing a favorable cost-effectiveness ratio for GC prevention (14). Emerging evidence indicates that the proton pump inhibitors (PPIs)’s long-term use may lead to GC risk, presenting new challenges in prevention and treatment strategies (12). Further research is needed to optimize drug use and inform clinical guidelines effectively. However, these studies suffer from issues such as methodological heterogeneity, differences in population characteristics, and inconsistent classification criteria for risk factors, resulting in significant contradictions among the results of different meta - analyses. This dispersion of evidence seriously hinders the formulation of clinical decisions and prevention strategies.

Although several meta-analyses of observational studies have investigated various risk factors for GC, differences in research design, exposure assessments, and outcomes have complicated the drawing of definitive conclusions. It is worth noting that although some systematic reviews have explored the association between specific risk factors and GC, there is currently a lack of research that uses the umbrella review method to hierarchically integrate existing evidence. Before effective prevention strategies can be formulated, it is crucial to systematically assess the quality, potential biases, and validity of the existing studies on GC risk factors. To address this gap, we conducted an umbrella review to consolidate the available evidence on the risk factors for GC.

## Methods and analysis

### Design and registration

Systematic literature searching, data extraction, and studies’ analysis focusing on GC risk factors were conducted. The process followed Preferred Reporting Items for Systematic Reviews and Meta-Analyses (PRISMA) guidelines (24). This study adhered to the methodological principles outlined in the Joanna Briggs Institute Manual for Evidence Synthesis of Umbrella Reviews (25), as well as the procedures detailed in the Cochrane Handbook (26). Additionally, the review was registered with the International Prospective Register of Systematic Reviews (PROSPERO) under registration number CRD42023447199 (https://www.crd.york.ac.uk/PROSPERO/).

### Eligibility criteria

Meta-analyses assessing GC risk factors across any ethnicity, sex, country, or setting were eligible for inclusion. If a single meta-analysis reported multiple risk factors, data on each risk factor were extracted separately. In cases where several meta-analyses (published more than 24 months apart) evaluated the same risk factor, we included the most recent one for analysis. For studies published within 24 months, priority was given to the meta-analysis with the most prospective cohorts. If the number of cohorts was the same, preference was given to the study with the higher AMSTAR score (27, 28). Study quality was independently assessed by two reviewers using AMSTAR-2 criteria. Discrepancies in scoring were resolved through iterative re-examination of the original articles followed by consultation with a third senior researcher when necessary. Moreover, if the latest meta-analysis did not perform a dose-response analysis, while another did, both were included for data extraction. Meta-analyses focusing on therapeutic interventions for GC, as well as non-English studies, animal studies, and cell culture studies, were excluded.

### Population

This study systematically examines meta-analyses assessing risk factors for GC. The primary focus of the included studies was to identify factors that influence the risk of GC, either by increasing or reducing it. Studies on the efficacy of treatments for GC, pathogenesis of GC, and factors related to GC exacerbation or recurrence were excluded.

### Exposure

Meta-analyses that identified at least one risk factor for gastric cancer (GC) were included in this review. These factors covered a broad range, including environmental, lifestyle, disease-related, treatment-related, demographic, genetic, social, and psychophysiological aspects. These risk factors was evaluated through the use of odds ratios (OR), relative risks (RR), or hazard ratios (HR), accompanied by 95% confidence intervals (CIs).

### Outcomes

The diagnosis of GC in the included studies must follow internationally accepted guidelines, such as the European Society for Medical Oncology (ESMO) Clinical Practice Guidelines, which outline the diagnostic, treatment, and follow-up protocols for patients with GC (29).

### Study designs

Only meta-analyses of studies assessing GC risk factors across various ethnicities, sexes, countries, or settings were eligible for inclusion. Studies focused on GC risk factors and provided comprehensive descriptions of their methods, including the search strategy, inclusion and exclusion criteria, quality assessment, result evaluation, analysis procedures, and criteria for interpreting the results. The original studies included in the reviews consisted of prospective or retrospective cohort studies, case-control studies, or cross-sectional studies.

### Information sources

A systematic search was performed in PubMed, Embase, Web of Science, and the Cochrane from the establishment of the database to April 2024 to find relevant meta-analyses, including both interventional and non-interventional studies. Additionally, we reviewed the reference lists to locate other relevant articles.

### Search strategy

These databases were searched using Medical Subject Headings (MeSH), keywords, and text terms associated with GC, in accordance with the Scottish Intercollegiate Guidelines Network (SIGN) recommendations for literature search methodology: (((risk) OR (incidence)) AND ((systematic review) OR (meta-analysis))) AND (((gastric cancer) OR (stomach cancer)) OR (stomach neoplasms)) (30).

### Study selection

**Figure 1:**
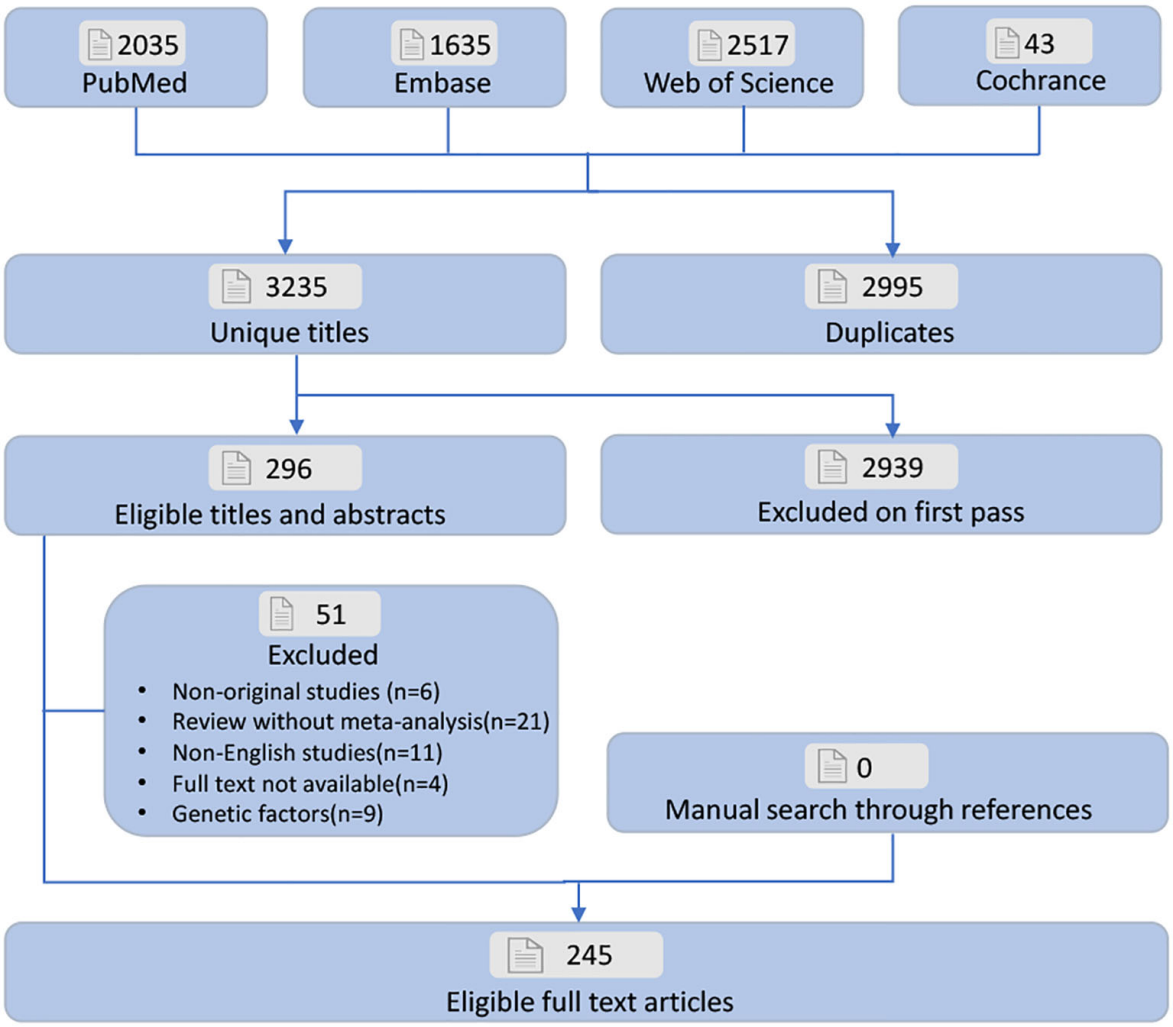
Flowchart of the systematic search and selection process.

All studies identified were initially screened with EndNote X9. After eliminating duplicates, two authors independently reviewed the titles and abstracts to select meta-analyses that met the inclusion criteria, followed by a full-text review. Any discrepancies between the two authors were resolved by a third author. Furthermore, a manual search of reference lists was conducted to identify additional meta-analyses that may have been overlooked (**Figure 1**).

### Assessment of methodological quality

Two reviewers independently assessed the methodological rigor, employing the AMSTAR instrument, which is a well-established and dependable method for evaluating the quality of meta-analyses (27, 31). The strength of epidemiological data supporting each risk factor was graded into one of four categories: Class I (indicating strong evidence), Class II (suggesting strong evidence), Class III (indicating evidence), Class IV (suggesting limited evidence), and NS (indicating no significant evidence), as detailed in **Table 1** (32–34).

**Table 1:** Evidence classification criteria.

Evidence class	Description
Class I: convincing evidence	>1000 cases (or >20,000 participants for continuous outcomes), statistical significance at *P* < 10^−6^ (random-effects), no evidence of small-study effects and excess significance bias; 95% prediction interval excluded the null, no large heterogeneity (I^2^ < 50%)
Class II: highly suggestive evidence	>1000 cases (or >20,000 participants for continuous outcomes), statistical significance at *P* < 10^−6^ (random-effects) and largest study with 95% CI excluding the null value
Class III: suggestive evidence	>1000 cases (or >20,000 participants for continuous outcomes) and statistical significance at *P* < 0.001
Class IV: weak evidence	The remaining significant associations with *P* < 0.05
NS: non-significant	*P* > 0.05

### Data extraction

Two researchers separately assessed the eligible studies to collect relevant data, such as the names of the authors, publication years, risk factors, types of gastric cancer, sample sizes, number of cases, total number of participants, study designs (e.g., cross-sectional, case-control, cohort), durations of follow-up, and risk measures (including RR, OR, HR, etc.) along with their 95% confidence intervals. The methods used for meta-analysis (whether random or fixed effects), heterogeneity evaluations (I², Cochran’s Q), and assessments of publication bias (through Egger’s and Begg’s tests, as well as funnel plots) were also recorded. For studies involving dose-response relationships or subgroup analyses, we noted P-values concerning nonlinearity and subgroup results. Any discrepancies were addressed by consulting another author. All papers contain reported data from unrelated individuals.

### Data summary

RR, OR, or HR along with their 95% CIs were re-estimated by applying either random or fixed-effects models. Additionally, we evaluated heterogeneity (using I² and Cochran’s Q-test) and the potential for small-study effects (via Egger’s or Begg’s tests) in meta-analyses, provided that the analysis involved more than 10 studies and sufficient data were available (35–37). Heterogeneity testing and publication bias detection were performed using Review Manager v5.4.1 (Cochrane Collaboration, Oxford, UK) and Stata v15.0, respectively. The risk factors were categorized into five distinct groups according to the health ecological model (38, 39): individual characteristics (such as age, gender, genetic factors, birth status, height, weight, BMI, pre-existing conditions, and prior treatments), lifestyle choices (including diet, physical activity, smoking, alcohol consumption, sleep habits, and working hours), social relationships (such as marital status, family dynamics, and social interactions), economic variables (like occupation, household income, and debt), and environmental influences (including urban vs. rural location, presence of pets, immigration status, and living conditions). When the OR, RR, or HR value of a factor is greater than 1 and there is a significant statistical difference, we consider this factor to be a risk factor; On the contrary, it is a protective factor for the stomach.

For risk factors classified as Class I or II evidence, a sensitivity analysis was performed when adequate data were available, in order to assess the individual studies’ impact on the overall evidence strength. We also conducted a dose-response analysis for GC risk factors based on the meta-analyses included in the review. Additionally, if the latest meta-analysis did not incorporate clinical studies from other reviews, we pooled data from these studies and carried out a new analysis. Heterogeneity tests were considered significant with a P-value of < 0.10, while other statistical tests were deemed significant at a *P*-value of < 0.05. Evidence synthesis was conducted using Review Manager v5.4.1 (Cochrane Collaboration, Oxford, UK), while sensitivity analysis and the Egger and Begg tests were performed using Stata v15.0.

### Major outcomes

#### Meta-analyses characteristics

Literature searching and selection was depicted in **Figure 1**. 6,230 unique articles were identified. 245 meta-analyses (7, 9–17, 21–23, 40–271) met the inclusion criteria. We extracted 117 distinct risk factors, consisting of 77 significantly associated and 40 non-significantly associated factors (**Supplementary Table S1**). Our analysis identified 42 adverse and 35 favorable associations with statistical significance. After a comprehensive quality assessment using established guidelines, most outcomes were categorized as Class IV (low quality) or NS (non-significant) evidence. Notably, only 17 risk factors (14.5%) were classified as Class I or II evidence. Among the identified factors, innate personal traits and behavioral lifestyles emerged as the primary contributors to GC risk.

#### Class I evidence

##### Cruciferous vegetable

**Figure 2:**
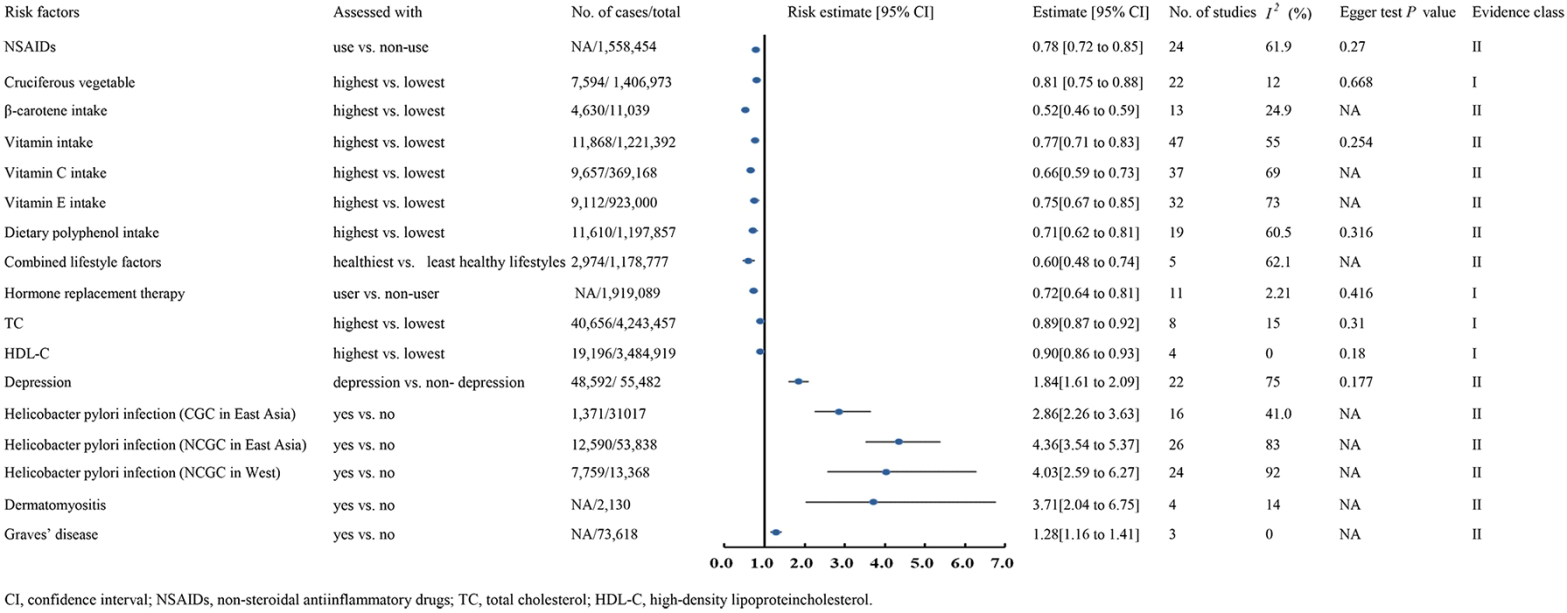
Forest plots of GC risk factors for class I and class II evidence.

A meta-analysis including 6 cohorts and 16 case-control studies, with 1,406,973 patients and 7,594 cases, showed that the cruciferous vegetables consumption can significantly decrease GC risk(RR 0.81, 95% CI 0.75-0.88) (21) (**Figure 2**).

##### High-density lipoprotein cholesterol

A meta-analysis involving 4 cohorts, comprising 3,484,919 participants and 19,196 cases, demonstrated that higher HDL-C were linked to lower GC risk (HR 0.90, 95% CI 0.86-0.93) (9) (**Figure 2**).

### Total cholesterol

A meta-analysis encompassing 8 cohorts, with 4,243,457 participants and 40,656 cases, reported that elevated TC levels were linked to a lower GC risk (HR 0.89, 95% CI 0.87-0.92) (9) (**Figure 2**).

### Hormone replacement therapy

A meta-analysis with 7 cohorts and 4 case-control studies, comprising 1,919,089 participants, found a significant association between estrogen-based hormone replacement therapy and a reduced GC risk (RR 0.72, 95% CI 0.64-0.81) (245) (**Figure 2**).

### Class II evidence

#### Innate personal trait

A meta-analysis of 22 studies, including 2 cohorts and 22 case-control studies, reported that depression increases the GC risk (OR 1.84, 95% CI 1.61-2.09) (259). Similarly, a meta-analysis of 4 case-control studies with 2,130 participants revealed a significant link between dermatomyositis and elevated GC risk (SIR 3.71, 95% CI 2.04-6.75) (236). In comparison, a meta-analysis of three studies involving 73,618 participants showed that individuals with Graves’ disease had a higher GC risk than healthy populations (SIR 1.28, 95% CI 1.16-1.41) (236). Conversely, a meta-analysis of 24 studies with 1,558,454 patients found NSAID use significantly reduced GC risk (RR 0.78, 95% CI 0.72-0.85) (169). Moreover, a meta-analysis of 26 studies demonstrated that *Hp* infection significantly raised GC risk (RR 4.36, 95% CI 3.54-5.37) (14) (**Figure 2**).

### Behavioural lifestyles

A meta-analysis of 47 studies, involving 11 randomized controlled trial, 7 cohorts and 29 case-control studies, comprising 1,221,392 participants and 11,868 cases, reported that higher vitamin intake was associated with a lower risk of GC compared to lower intake (RR 0.77, 95% CI 0.71-0.83) (112). The analysis further demonstrated that increased intake of vitamin C (RR 0.66, 95% CI 0.59-0.73) and vitamin E (RR 0.75, 95% CI 0.67-0.85) reduced GC risk (112). Regarding dietary polyphenol intake, a meta-analysis of 7 cohort and 12 case-control studies involving 1,197,857 participants revealed that individuals with higher polyphenol consumption had a significantly lower GC risk (RR 0.71, 95% CI 0.62-0.81) (240). Similarly, a large-scale meta-analysis of 13 case-control studies found that higher β-carotene intake significantly reduced GC risk (OR 0.52, 95% CI 0.46-0.59) (162). Moreover, a meta-analysis of 5 cohort studies concluded that healthier combined lifestyle factors significantly decreased GC risk (OR 0.60, 95% CI 0.48-0.74) (220) (**Figure 2**).

### Class III evidence

**Figure 3:**
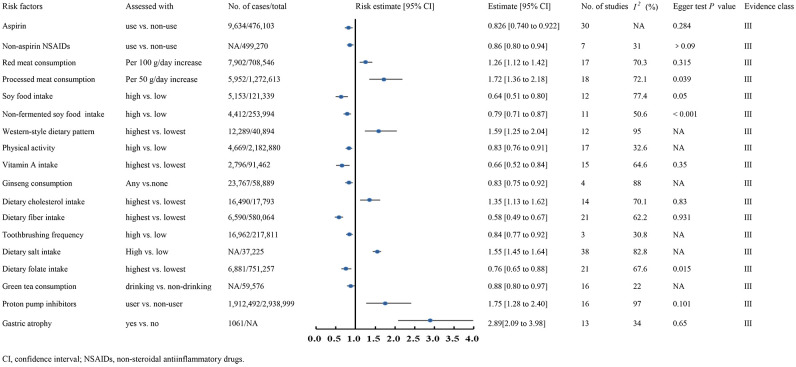
Forest plots of GC risk factors for class III evidence.

Class III evidence demonstrated that aspirin use (RR 0.826, 95% CI 0.740-0.922) (210), non-aspirin NSAID use (RR 0.86, 95% CI 0.80-0.94) (169), higher soy food intake (RR 0.64, 95% CI 0.51-0.80) (233), non-fermented soy food intake (RR 0.79, 95% CI 0.71-0.87) (233), increased physical activity (RR 0.83, 95% CI 0.76-0.91) (22), higher vitamin A intake (RR 0.66, 95% CI 0.52-0.84) (144), ginseng consumption (RR 0.83, 95% CI 0.75-0.92) (152), higher dietary fiber intake (OR 0.58, 95% CI 0.49-0.67) (107), frequent toothbrushing (OR 0.84, 95% CI 0.77-0.92) (235), higher folate intake (OR 0.76, 95% CI 0.65-0.88) (171), and increased green tea consumption (OR 0.88, 95% CI 0.80-0.97) (212) were associated with reduced GC risk (**Figure 3**).

Conversely, EB virus infection (OR 18.57, 95% CI 15.69-21.98) (215), higher red meat consumption (RR 1.26, 95% CI 1.12-1.42) (193), processed meat consumption (RR 1.72, 95% CI 1.36-2.18) (193), intestinal metaplasia (RR 5.16, 95% CI 3.28-5.16) (224), gastric atrophy (RR 2.89, 95% CI 2.09-3.98) (261), a western-style dietary pattern (OR 1.59, 95% CI 1.25-2.04) (97), higher dietary cholesterol intake (OR 1.35, 95% CI 1.13-1.62) (229), increased dietary salt intake (OR 1.55, 95% CI 1.45-1.64) (256), and proton pump inhibitor use (OR 1.75, 95% CI 1.28-2.40) (12) were linked to elevated GC risk (**Figure 3**). Dose-response analysis revealed that every 100g increase in daily red meat intake raised GC risk by 26%, while each 50g increase in processed meat intake per day elevated the risk by 72%.

### Class IV and NS evidence

This umbrella review identified 40 class IV evidence-based risk factors, including 13 protective and 27 risk factors. The top ten protective factors were refrigerator use (OR 0.70, 95% CI 0.56-0.88) (265), higher allium vegetable intake (RR 0.78, 95% CI 0.67-0.91) (141), greater garlic consumption (OR 0.65, 95% CI 0.49-0.87) (244), adherence to a healthy dietary pattern (OR 0.69, 95% CI 0.53-0.89) (97), the Mediterranean dietary score (MDS) (OR 0.69, 95% CI 0.53-0.90) (209), the dietary inflammatory index (DII) (RR 0.63, 95% CI 0.45-0.88) (209), metformin use (RR 0.84, 95% CI 0.73-0.96) (253), statin use (OR 0.74, 95% CI 0.67-0.80) (16), higher tomato product consumption (OR 0.73, 95% CI 0.60-0.90) (104), and increased selenium levels (OR 0.87, 95% CI 0.78-0.97) (151).

The top ten risk factors with the highest effect values were MAFLD (RR 1.49, 95% CI 1.17-1.91) (7), pernicious anemia (OR 6.8, 95% CI 2.6-18.1) (102), frequent consumption of refined grains (≥3 times/week vs. <3 times/week) (OR 1.63, 95% CI 1.49-1.79) (216), papillomavirus infection (OR 5.80, 95% CI 3.27-10.31) (221), higher educational attainment (OR 2.97, 95% CI 1.93-4.58) (101), combined socioeconomic position (OR 2.64, 95% CI 1.05-6.63) (101), current cigarette smoking (OR 1.61, 95% CI 1.49-1.75) (212), insulin use (RR 1.65, 95% CI 1.02-2.68) (95), inflammatory myopathies (SIR 2.68, 95% CI 1.40-5.12) (236), and higher chili consumption (OR 1.51, 95% CI 1.02-2.00) (228).

Additionally, this umbrella review identified 40 non-significant risk factors, with detailed data provided in **Supplementary Table S1**.

### Heterogeneity

In this study, 59% of risk factors were reanalyzed using random- or fixed-effects models, revealing significant heterogeneity in approximately 63% (I² > 50% or Cochran’s Q-test P < 0.1). Heterogeneity in most outcomes was likely influenced by factors such as study setting, geographical region, ethnicity, gender, age, sample size, study design, follow-up duration, and adjustments for confounding variables. Among the remaining 41% of risk factors, 56% exhibited considerable heterogeneity, while 2.6% did not report heterogeneity results.

### Assessment of risk of bias

In the reassessment, publication bias was evaluated for 56.2% of the identified risk factors using Egger’s test, which detected bias in nine factors. For outcomes not reanalyzed, statistical tests or funnel plots revealed publication bias in 5.1% of the risk factors. The remaining outcomes showed no significant evidence of publication bias or lacked bias assessment.

### AMSTAR score

The median AMSTAR score for all identified risk factors was 8 (range 6-10). Detailed AMSTAR scores for each outcome are listed in **Supplementary Table S2**.

## Discussion

### Principal findings and possible explanations

Gastric cancer remains a common malignancy worldwide, ranking third in cancer-related mortality. In China, the burden of GC is particularly severe, with an incidence rate of 34.6 per 100,000 and a mortality rate of 30.2 per 100,000 (267). The etiology of GC is multifaceted, involving intricate interactions among genetic, environmental, and lifestyle factors, many of which remain poorly understood. Despite extensive research, several risk factors contributing to GC have not received adequate attention (268). Current studies categorize GC risk factors into demographic, socioeconomic, environmental, infectious, genetic, drug-related, and psychological factors. A subset of these risk factors is modifiable, prompting some researchers to classify GC as “one of the preventable cancers” (269). Over the past decades, clinical and evidence-based studies have explored these risk factors extensively, using systematic reviews and meta-analyses to synthesize findings from diverse populations. This umbrella review aims to evaluate the strengths and limitations of existing evidence from systematic reviews and meta-analyses on GC risk factors, providing a comprehensive understanding of the potential contributors to its development and progression. By synthesizing insights from systematic reviews and meta-analyses, this review offers a robust theoretical foundation for the design of more effective prevention and control strategies and highlights priorities for future research. This umbrella review identified 117 unique risk factors for GC, including 77 factors significantly associated with GC and 40 with non-significantly associations. Among the significant risk factors, 42 were adverse while 35 were favorable. Following a rigorous quality assessment using established classification criteria, most outcomes were classified as Class IV or NS evidence. Only 17 risk factors (14.5%) met the criteria for Class I or II evidence. Notably, personal traits and behavioral lifestyles were the primary contributors to the risk of GC.

### Dietary factors play an important role in the occurrence and development of gastric cancer

This study observed a significant negative correlation between cruciferous vegetable intake and the risk of GC (Class I evidence). Supporting this finding, Fang et al. (270) analyzed 76 cohort studies and reported an inverse relationship between fruit and white vegetable consumption and gastric cancer risk. However, a Japanese cohort study found no significant association between fresh fruit and vegetable intake and gastric cancer risk (271). A hypothesis suggests that a protective threshold exists for vegetables and fruits consumption, beyond which no additional benefit occurs (272). This discrepancy may also arise from contextual factors: the Japanese diet traditionally includes high salt intake (e.g., pickled vegetables and soy sauce), which could counteract the benefits of fresh vegetables. Additionally, variations in Helicobacter pylori infection rates, genetic susceptibility, or differences in vegetable preparation methods (e.g., fermentation vs. raw consumption) might contribute to conflicting results. Future studies should account for dietary patterns holistically and adjust for salt intake as a potential confounder to clarify these associations. Notably, the studies reviewed here did not establish a significant dose-response relationship between vegetable intake and gastric cancer risk, highlighting the need for further investigation. Additionally, a meta-analysis by Ren et al. (265, 273) among Korean and Chinese populations found that consuming pickled vegetables increased the risk of gastric cancer by 50%, while refrigerator use reduced cancer risk. This implies that processed vegetables, with their high salt and sugar content, may counteract the protective effects of fresh vegetables.

### Vitamin Effects

This study indicates that the intake of vitamins, including vitamin C (Class II evidence), vitamin E (Class II evidence), and vitamin A (Class III evidence), is inversely correlated with GC risk. Vitamin C, a water-soluble antioxidant and enzyme cofactor, is present in various animal and plant sources. It exists in two forms: reduced (ascorbic acid, AA) and oxidized (dehydroascorbic acid, DHA), with AA being the predominant form in the human body, essential for normal physiological functions in many organisms (274). As an antioxidant and free radical scavenger, vitamin C plays a key role in collagen synthesis. Moderate intake may help reduce tissue or DNA damage (275). Vitamin C has been shown to disrupt the microenvironment created by bacteria, enhance the diffusion of antibiotics into the gastric mucosa, inhibit *Hp* colonization and growth, and thereby reduce the risk of progression of precancerous lesions, modifying *Hp*’s effect on gastric cancer (270, 276). In 2007, experts from the American Cancer Institute suggested that high intake of vitamin C-rich foods may enhance anti-cancer effects in GC patients (277). An ecological study in Poland found a significant negative correlation between high intake of vegetables, fruits, and vitamin C and GC incidence. Experiments indicated that increasing vitamin C-rich food intake may help prevent GC development (278). A meta-analysis found that daily Vitamin C intake reduced GC risk by 26% (279). However, controversies remain regarding its protective role, as some studies report limited or nonsignificant effects in specific subpopulations (280). The precise role of vitamin C in GC and its potential as a diagnostic marker, including the significance of serum vitamin C levels, remain uncertain.

Vitamin E, a fat-soluble antioxidant, protects against oxidative stress, lipid peroxidation, and tumor cell proliferation. It exerts a protective effect on the gastric mucosa. Vitamin E also neutralizes nitroso ions, inhibits nitrosamine production, activates cellular immunity, suppresses humoral immunity, prevents tumor cell proliferation, and induces tumor cell apoptosis (281, 282). Studies have shown that vitamin E can protect DNA from oxidative damage and prevent potential gene mutations, thereby playing an anti-cancer role (283). In a prospective cohort study by Egnell et al. (284), follow-up of the trial population revealed that increasing total vitamin E intake in adults reduces the risk of gastrointestinal tumors. Although higher vitamin E intake reduces the risk of GC, its effects vary by anatomical site and histological subtype. Specifically, increased vitamin E intake lowers the risk of non-cardia gastric cancer but shows no significant association with diffuse gastric cancer. A meta-analysis of dietary vitamin E intake, involving 24 studies with 7,095 participants, examined the relationship between dietary vitamin E and GC risk. Comparing the highest and lowest doses of vitamin E intake, the study found significant heterogeneity and indicated that vitamin E’s effects differ across GC subtypes. However, it did not address the relationship between serum vitamin E levels and GC risk (285). Therefore, while increasing vitamin E intake may help reduce GC risk, the diagnostic significance and clinical value of serum vitamin E levels for GC remain uncertain and warrant further investigation.

Similarly, β-carotene intake was associated with a significant reduction in gastric cancer risk (Class II). Consistent with these findings, a Chinese meta-analysis also supports the inverse association between tomato consumption and gastric cancer risk (162). In a Finnish cohort study, carotenoids did not affect the risk of cardia cancer but significantly reduced the risk of non-cardia gastric cancer. They inhibit carcinogen synthesis, reduce carcinogen activity, and exhibit anti-mutagenic and anti-teratogenic effects (286). *In vitro* and *in vitro* studies have also confirmed the anti-cancer and cancer-preventive properties of carotenoids and their extracts, with therapeutic efficacy positively correlated with dose (287). A study by Silvia et al. (288) in northern Italy found that higher carotenoid intake was associated with a greater inhibition of GC cells.

### The impact of underlying diseases on the risk of gastric cancer cannot be ignored

#### Psychological factors

This study highlights the significant influence of depression and psychological distress on GC risk. Depression increases the risk of GC by 84% compared to non-depressed individuals (Class II). Under the modern bio-psycho-social model, the influence of psychological factors on cancer incidence has gained increasing attention. Research has shown that depression raises the risk of gastric adenoma or GC by 4.54 times (OR: 4.54; 95% CI: 2.42-8.55) (289). In a Japanese cross-sectional survey involving 29,926 GC patients, only 36% of individuals with severe psychological stress participated in cancer screening, underscoring the negative impact of psychological stress on screening uptake (290). Chronic psychological distress and stress disrupts the hypothalamus-pituitary-adrenal axis, leading to endocrine disruptions that impair immune cells activity, including natural killer(NK) cells, T cell subsets, and B cells, thereby increasing GC susceptibility (291). Psychological distress also increases the production of reactive oxygen species (ROS). Excessive ROS production due to psychological distress can activate the ABL proto-oncogene 1 (ABL1), promoting inflammatory pathways and GC progression (292). Psychological distress is common among GC patients at all stages and is associated with poor prognosis (293). These findings emphasize the need for integrating mental health interventions in GC prevention strategies.

#### *Helicobacter pylori* infection

This study corroborates previous findings, confirming that *Helicobacter pylori* infection significantly increases the risk of GC (Class II evidence). A recent 10-year prospective study showed that gastric mucosal atrophy and intestinal metaplasia are reversible after *Hp* eradication. In this study, 65 participants were *Hp*-negative, and 533 were *Hp*-positive (442 in the eradication group and 91 in the non-eradication group). After 1 year of follow-up, the *Hp*-positive eradication group exhibited significant improvement in gastric mucosal atrophy. The differences in gastric antral intestinal metaplasia were eliminated after 5 years, and those in gastric body intestinal metaplasia were eliminated after 3 years (294). Studies have shown that *Hp* eradication is especially beneficial for asymptomatic patients and those after endoscopic early cancer resection, reducing the gastric cancer risk by 34% (295). In first-degree relatives with a family history of gastric cancer and Hp infection, the risk of gastric cancer was reduced by 55% following *Hp* eradication (296). *Hp* is a key controllable risk factor for gastric cancer, particularly in patients with a family history. Early eradication significantly reduces gastric cancer risk. Widespread *Hp* screening offers promising prospects for gastric cancer prevention and treatment.

#### The use of certain drugs may reduce the risk of gastric cancer

This study found that long-term NSAID use significantly reduces GC risk (Class II evidence), with both aspirin and non-aspirin NSAIDs demonstrating protective effects (Class III evidence). NSAIDs primarily exert anti-tumor effects by inhibiting COX, a key enzyme in PG synthesis. This inhibition reduces COX-derived PGE2 production, which is closely linked to GC development. Possible mechanisms include: 1) Echizen et al. (297) identified that the interaction between the TLR/MyD88 and COX-2/PGE2 pathways plays a key role in tumor microenvironment formation. NSAIDs inhibit COX-2, reducing PGE2 levels and disrupting the signal transduction of both pathways, thereby exerting anti-tumor effects. 2) PGE2 suppresses macrophage and natural killer cell activity, decreases the production of lymphokines such as tumor necrosis factor-α, interferon-γ. 3) Gene mutations such as those in APC, ras, and p53 lead to increased COX-2 expression, enhanced PGE2 synthesis, and promote tumor cell proliferation. In contrast, NSAIDs reduce PGE2 synthesis and inhibit tumor cell growth. However, NSAIDs do not solely rely on the COX-2 pathway to suppress tumor cell growth. β-catenin, a key regulator of cell adhesion, is influenced by the APC gene. As a tumor suppressor, APC regulates β-catenin’s adhesion function. When APC is mutated, β-catenin dissociates from APC, activating the expression of oncogenes like c-myc and cyclin D1, thereby promoting tumor cell growth. Liggett et al. (298) demonstrated that NSAIDs downregulate β-catenin and reduce Smad2/3 transfer protein complex activity, inhibiting tumor cell proliferation, adhesion, and growth. Akrami et al. (299) investigated the anti-tumor effects of NSAIDs on human gastric cancer cells (AGS) and found that NSAIDs upregulate p53 expression, induce tumor cell apoptosis, and inhibit tumor onset and progression.

This study demonstrates that estrogen replacement therapy (ERT) significantly reduces the risk of GC (Class I evidence). While gastric cancer is typically regarded as an estrogen-independent tumor, in contrast to breast and endometrial cancers, emerging evidence suggests that estrogen may play a protective role in its progression (300, 301). The incidence of gastric cancer is higher in men compared to premenopausal women (2-3:1), but it increases in postmenopausal women (1, 2). An analysis of the relationship between estrogen exposure and GC risk indicates that ERT decreases the incidence of GC, while anti-estrogen therapies may increase the risk (302). Estrogen also exhibits potential immunomodulatory effects within the tumor microenvironment (303). Reyes-Ramos et al. (304) reported that ERα is expressed on both GC cells and macrophages within the tissue microenvironment. Estrogen interacts with these macrophages, modulating their activation, recruitment, and chemokine secretion, which collectively influencing tumor progression. However, since ERT is primarily used in postmenopausal women, its precise role in reducing GC risk requires confirmation through further prospective studies.

### Blood lipid-related indicators may be significantly associated with the risk of gastric cancer

#### TC Factors

This study found that higher levels of TC (Class I evidence) and HDL-C (Class I evidence) are associated with a reduced risk of GC. Serum TC levels are crucial for cell structure and function, playing a key role in maintaining the structure and activity of biological membranes (305). Changes in TC levels can affect various cell functions, including enzyme activity, endocytosis, and receptor function (306). In some malignancies, elevated serum TC levels promote tumor progression, such as in testicular, prostate, and colorectal cancers (307). In contrast, reduced serum TC levels can accelerate tumor progression in other malignancies, including GC, hepatocellular carcinoma, intrahepatic bile duct carcinoma, and pancreatic cancer (308). The findings suggest that serum TC levels play a complex role in the occurrence and progression of malignant tumors. TC serves as a precursor for several biochemical pathways involved in the synthesis of key signaling molecules, such as vitamin D and steroid hormones, which are implicated in the etiology of certain malignancies (309). Studies have shown that long-term serum TC deficiency can activate nuclear factor κB, a key transcription factor involved in regulating immunity, inflammation, apoptosis, carcinogenesis, and other processes (310) Additionally, low serum TC levels may deplete CD8+ T cells in the tumor microenvironment, weakening the immune system’s protective function (311, 312). These factors may explain the negative correlation between serum TC levels and gastric cancer, although the underlying mechanisms require further investigation.

### Blood lipid components

The mechanisms underlying the correlation between blood lipid components and GC include both anti-tumor and carcinogenic pathways. HDL-C exhibits anti-tumor effects through its reverse cholesterol transport function, antioxidant, and anti-inflammatory properties, while LDL-C may contribute to carcinogenesis by impairing immune system function. A key function of HDL-C is reverse cholesterol transport. HDL-C binds to cell surface receptors, promoting the efflux of intracellular cholesterol to the liver and steroidogenic cells for metabolism, thereby maintaining normal intracellular cholesterol balance (313). Elevated HDL-C levels may lower peripheral cholesterol, thereby inhibiting tumor development and lymph node metastasis (314). Furthermore, the antioxidant properties of HDL-C contribute to its anti-tumor role. HDL-C can neutralize harmful oxidants, preventing oxidative stress-induced DNA damage, which otherwise promotes tumorigenesis and tumor transformation (315).

This study proposes a tiered prevention strategy for gastric cancer based on evidence levels: prioritizing the promotion of measures supported by Class I-II evidence, including increasing intake of cruciferous vegetables and vitamin C/E, improving high-density lipoprotein cholesterol, and standardizing Helicobacter pylori eradication treatment; Strictly control the main risk factors, such as implementing PHQ-9 scale screening and initiating cognitive-behavioral interventions for patients with depression, and regular gastroscopy monitoring for patients with autoimmune diseases. For protective factors such as dietary fiber and green tea, as well as risk factors such as red meat and high salt diet, it is recommended to develop a limit control plan based on individual metabolic characteristics. At the same time, it is called for to carry out multi center RCTs to verify potential protective effects such as beta carotene, and to construct a collaborative prevention and control system of gastroenterology psychiatry nutrition to optimize the prevention and treatment of gastric cancer.

### Research gaps and future directions

While this umbrella review consolidates extensive evidence, several gaps remain. First, most studies focused on Asian and Western populations, with limited data from Africa, South America, and indigenous communities, where genetic and environmental risk profiles may differ. Second, emerging exposures (e.g., microplastics, antibiotic overuse, and alterations in the gut microbiome) remain underexplored. Third, biomarker-based risk assessments (e.g., genetic polymorphisms, epigenetic markers, or metabolic signatures) are rarely integrated with traditional epidemiological factors, limiting personalized prevention strategies. Additionally, interactions between psychological stress and biological pathways (e.g., immune dysfunction or oxidative stress) warrant deeper investigation. Future research should prioritize longitudinal studies in underrepresented populations, incorporate multi-omics approaches, and evaluate the cost-effectiveness of targeted interventions (e.g., H. pylori eradication in high-risk subgroups). Addressing these gaps will enhance our ability to mitigate the global burden of gastric cancer through precision prevention.

## Limitations and strengths

This study has several limitations. Firstly, we only searched English-language databases, which may have introduced publication bias and language bias by excluding studies published in other languages. Secondly, we included only published data, excluding unpublished or forthcoming evidence. Third, data for this study were extracted and analyzed directly from systematic reviews and meta-analyses, excluding original studies not included in these reviews. In addition, this study included more retrospective studies than RCTs, with limited evidence levels. Despite these limitations, this umbrella review provides the first comprehensive summary of existing evidence from previous meta-analyses on gastric cancer risk factors. This umbrella review evaluated the strengths and weaknesses of the current evidence from systematic reviews and meta-analyses on risk factors for GC. It presents a thorough understanding of potential factors influencing the onset and progression of GC, lays the groundwork for the development of more effective prevention and control strategies, and proposes avenues for future clinical research. The study adhered to stringent systematic procedures, with two independent authors performing literature searches, selecting relevant studies, and extracting data. When adequate data were available, we reanalyzed the RR, OR, or HR using 95% CIs and applying either random or fixed-effects models. We evaluated heterogeneity and assessed publication bias for each included meta-analysis. Furthermore, we utilized two established approaches-AMSTAR and evidence classification criteria-to assess methodological quality and categorize the evidence for each risk factor. They reduce subjective bias and improve the accuracy and reliability of evidence assessment by providing standardized and objective frameworks. This helps to make informed decisions in clinical practice and policy-making. It also promotes global research exchange and cooperation, and can guide future research directions by highlighting areas with insufficient evidence. However, these standards are not without flaws. Their applications may be subjective, and different standards may lead to different classifications. They may have overlooked the diversity of evidence and not fully considered various research backgrounds and non-traditional sources of evidence. In addition, they often lag behind the rapid development of medical research and run the risk of over reliance on high-level evidence, which may overlook valuable information from low-level research.

## Conclusion

This umbrella review identified 77 risk factors significantly associated with GC, including 42 risk factors and 35 protective factors. Most were linked to innate traits and behavioral lifestyles. After assessing evidence quality, only 17 risk factors were classified as Class I or II. Protective factors included cruciferous vegetable intake, TC, HDL-C, NSAIDs, β-carotene, vitamin intake, and dietary polyphenol intake. Key risk factors comprised depression, *Helicobacter pylori* infection, dermatomyositis, and Graves’ disease. These findings provide a foundation for developing improved prevention strategies and treatments to reduce GC incidence, slow progression, and alleviate the global burden of GC-related diseases. However, current research has the drawback that the analysis of traditional epidemiological factors has not yet been systematically integrated with genetic and molecular markers. Future overall reviews should focus on combining genetic and molecular risk markers with traditional epidemiological factors. Future umbrella reviews should focus on integrating genetic and molecular risk markers alongside traditional epidemiological factors.

## Data Availability

Publicly available datasets were analyzed in this study. This data can be found here: PubMed, Embase, Web of Science, and the Cochrane.
