# Supplementary material for: Risk factors for gastric cancer: an umbrella review of systematic reviews and meta-analyses
Source: Front Oncol. 2025 Jun 26;15:1564575. doi: 10.3389/fonc.2025.1564575 (PMC12241803; doi:10.3389/fonc.2025.1564575)
Supplement: Supplementary file 1 [file Table1.docx]

| Supplementary Table S1. Risk factors for gastric cancer. | | | | | | | | | | | | | | | | | | | |
| --- | --- | --- | --- | --- | --- | --- | --- | --- | --- | --- | --- | --- | --- | --- | --- | --- | --- | --- | --- |
| Exposure | | Evidence class | | | Exposure Contrast | N^ | Included meta-analysis | | Sample Size | Largest Study^#^ | | Meta-analysis metric | | | Estimates [95% CI]^φ^ | No. of studies  (T/R/C/P)^§^ | Effects model | I^2^; Q test P value | Egger test P value |
| ***Significant associations*** | | | | | | | | | | | | | | | | | | | |
| EB virus | | | III | infection vs. non- infection | | 2 | Tavakoli 2020 | 4,116/4,116 | | | NA | | OR | 18.57[15.69 to 21.98] | | 20/0/0/20 | random | 55%; 0.001 | NA |
| NSAIDs | | | II | use vs. non-use | | 6 | Huang 2017 | NA/1,558,454 | | | 0.79(0.69,0.90) | | RR | 0.78 [0.72 to 0.85] | | 24/2/8/14 | random | 61.9%; 0.000 | 0.27 |
| Aspirin | | | III | use vs. non-use | | 12 | Niikura 2020 | 9,634/476,103 | | | NA | | RR | 0.826[0.740 to 0.922] | | 30/1/3/26 | random | NA | 0.284 |
| Non-aspirin NSAIDs | | | III | use vs. non-use | | 4 | Huang 2017 | NA/499,270 | | | 0.88(0.78,1.01) | | RR | 0.86[0.80 to 0.94] | | 7/0/2/5 | random | 31.0%;0.143 | ﹥0.09 |
| Red meat consumption | | | III | per 100 g/day increase | | 4 | Kim 2019 | 7,902/ 708,546 | | | 1.27(1.08,1.50) | | RR | 1.26 [1.12 to 1.42] | | 17/0/4/13 | random | 70.3%; 0.000 | 0.315 |
| Processed meat consumption | | | III | per 50 g/day increase | | 6 | Kim 2019 | 5,952/1,272,613 | | | 1.18(0.97,1.43) | | RR | 1.72[1.36 to 2.18] | | 18/0/7/11 | random | 72.1%; 0.000 | 0.039 |
| Intestinal metaplasia | | | III | IIM vs. CIM | | 2 | Du 2021 | NA/ 6,498 | | | 2.12(0.92,4.91) | | RR | 5.16 [3.28 to 8.12] | | 12/0/12/0 | fixed | 0%; 0.44 | NA |
| Fruit intake | | | IV | 100 g/day increase | | 9 | Naemi 2023 | 5,678/1,423,134 | | | 0.98(0.93,1.03) | | RR | 0.95 [0.90 to 0.99] | | 9/0/9/0 | random | 49%; 0.039 | NA |
| Vegetable and fruit intake | | | IV | 200 g/day increase | | 9 | Naemi 2023 | 4,475/1,244,753 | | | 0.92(0.86,0.99) | | RR | 0.94 [0.88 to 0.99] | | 5/0/5/0 | random | 37.6%;0.170 | NA |
| Allium vegetable intake | | | IV | highest vs. lowest | | 3 | Turati 2015 | 6,281/709,150 | | | 0.79(0.62,1.01) | | RR | 0.78 [0.67 to 0.91] | | 14/0/4/10 | random | 58.6%; 0.002 | 0.04 |
| Cruciferous vegetable | | | I | highest vs. lowest | | 1 | Wu 2013 | 7,594/ 1,406,973 | | | 0.84 (0.76–0.93) | | RR | 0.81 [0.75 to 0.88] | | 22/0/6/16 | fixed | 12.0%; 0.292 | 0.668 |
| Soy food intake | | | III | high vs. low | | 8 | Wang 2021 | 5,153/121,339 | | | 1.02(0.83,1.26) | | RR | 0.64 [0.51 to 0.80] | | 12/0/2/10 | random | 77.4%;< 0.001 | 0.050 |
| Fermented soy food intake | | | IV | high vs. low | | 4 | Wang 2021 | 7,256/308,498 | | | 1.01(0.88,1.06) | | RR | 1.19 [1.02 to 1.38] | | 15/0/8/7 | random | 65.8%; 0.000 | 0.170 |
| Non-fermented soy food intake | | | III | high vs. low | | 2 | Wang 2021 | 4,412/253,994 | | | 1.01(0.85,1.20) | | RR | 0.79 [0.71 to 0.87] | | 11/0/5/6 | random | 50.6%; 0.004 | < 0.001 |
| Garlic consumption | | | IV | any vs. none | | 6 | Huang 2022 | 3,299/63,801 | | | 0.81(0.58,1.14) | | OR | 0.65 [0.49 to 0.87] | | 11/1/2/8 | random | 69.8%; 0.000 | 0.490 |
| MAFLD | | | IV | yes *vs.* no | | 1 | Zou 2023 | 9,510/8,629,525 | | | 1.18(1.14,1.22) | | RR | 1.49 [1.17 to 1.91] | | 8/0/8/0 | random | 81.8%; 0.000 | 0.157 |
| Cholecystectomy | | | IV | yes *vs.* no | | 1 | Yang 2022 | 26,063/850,835 | | | 1.11(1.04,1.19) | | RR | 1.11 [1.03 to 1.20] | | 8/0/5/3 | random | 37.8%; 0.117 | 0.683 |
| Healthy dietary pattern | | | IV | highest *vs.* lowest | | 2 | Shu 2013 | 13,283/321,292 | | | 1.24(1.17,1.32) | | OR | 0.69 [0.53 to 0.89] | | 14/0/2/12 | random | 97.0%; 0.000 | NA |
| Western-style dietary pattern | | | III | highest *vs.* lowest | | 2 | Shu 2013 | 12,289/40,894 | | | 1.59(1.50,1.69) | | OR | 1.59 [1.25 to 2.04] | | 12/0/2/10 | random | 95.0%;0.0002 | NA |
| Alcohol-drinking dietary pattern | | | IV | highest *vs.* lowest | | 2 | Shu 2013 | 4,019/420,971 | | | 1.04(0.95,1.14) | | OR | 1.37 [1.11 to 1.70] | | 15/0/5/10 | random | 84.0%;0.004 | NA |
| MDS | | | IV | highest vs. lowest | | 3 | Moazzen 2020 | 1,611/530,183 | | | 0.85(0.69,1.05) | | OR | 0.69 [0.53 to 0.90] | | 3/0/2/1 | random | 68%; 0.04 | 0.01 |
| DII | | | IV | highest *vs.* lowest | | 3 | Moazzen 2020 | 1,546/519,542 | | | 0.84(0.67,1.05) | | RR | 0.63 [0.45 to 0.88] | | 4/0/2/2 | random | 66%; 0.03 | 0.26 |
| Physical activity | | | III | high vs. low | | 7 | Xie 2020 | 4,669/2,182,880 | | | NA | | RR | 0.83 [0.76 to 0.91] | | 17/0/12/5 | random | 32.6%;0.095 | NA |
| Pernicious anaemia | | | IV | yes vs. no | | 1 | Vannella 2013 | 7/453 | | | NA | | RR | 6.8 [2.6 to 18.1] | | 6/0/6/0 | random | 81.3%;< 0.0001 | 0.4 |
| Metformin | | | IV | use vs. non-use | | 2 | Seo 2022 | NA/1,195,462 | | | 0.99(0.99,0.99) | | RR | 0.84[0.73 to 0.96] | | 9/0/9/0 | random | 82.0%; 0.000 | NA |
| Tomato products consumption | | | IV | high vs. low | | 1 | Yang 2013 | 2,596/7,493 | | | NA | | OR | 0.73[0.60 to 0.90] | | 7/0/1/6 | random | 47.9%;0.07 | 0.04 |
| Body obesity^a^ | | | IV | obesity vs. normal weight | | 2 | Azizi 2023 | NA/13,946,947 | | | 1.19(1.14,1.24) | | RR | 1.22[1.07 to 1.39] | | 11/0/11/0 | random | 97.65%; NA | NA |
| Body over-weight^b^ | | | IV | overweight vs. normal weight | | 2 | Azizi 2023 | NA/14,015,733 | | | 1.13(1.10,1.16) | | RR | 1.16[1.05 to 1.27] | | 12/0/12/0 | random | 95.18%; NA | NA |
| Whole grains consumption | | | IV | intake vs. non-intake | | 5 | Wang 2020 | 1,428/3,891 | | | 0.92(0.82,1.03) | | OR | 0.87[0.79 to 0.95] | | 5/0/0/5 | random | 6.6%;0.369 | NA |
| Refined grains consumption | | | IV | ≥3times/wk vs.＜3times/wk | | 3 | Wang 2020 | NA | | | 1.58(1.30,1.91) | | OR | 1.63[1.49 to 1.79] | | 16/0/0/16 | fixed | 27.4%;0.138 | NA |
| Talc exposure | | | IV | any vs. none | | 1 | Chang 2020 | NA/112,002 | | | 1.49(1.28,1.74) | | RR | 1.21[1.03 to 1.42] | | 13/0/13/0 | random | 30.0%;0.14 | 0.35 |
| Crystalline silica exposure | | | IV | exposure vs. non- exposure | | 1 | Lee 2016 | NA | | | NA | | RR | 1.25[1.18 to 1.34] | | 50/0/31/19 | random | 74.3%; NA | <0.1 |
| β-carotene intake | | | II | highest *vs.* lowest | | 5 | Zhou 2016 | 4,630/11.039 | | | 0.46(0.34,0.62) | | OR | 0.52[0.46 to 0.59] | | 13/0/0/13 | Random | 24.9%;0.186 | NA |
| Vitamin intake | | | II | highest *vs.* lowest | | 1 | Kong 2014 | 11,868/1,221,392 | | | 0.72(0.63,0.83) | | RR | 0.77[0.71 to 0.83] | | 47/11/7/29 | random | 55.0%;0.000 | 0.254 |
| Vitamin A intake | | | III | highest *vs.* lowest | | 2 | Wu 2015 | 2,796/91,462 | | | 0.54(0.43,0.66) | | RR | 0.66[0.52 to 0.84] | | 15/0/2/13 | random | 64.6%;0.000 | 0.350 |
| Vitamin C intake | | | II | highest *vs.* lowest | | 1 | Kong 2014 | 9,657/369,168 | | | 0.60(0.51,0.70) | | RR | 0.66[0.59 to 0.73] | | 37/6/2/29 | random | 69.0%;0.000 | NA |
| Vitamin E intake | | | II | highest *vs.* lowest | | 3 | Kong 2014 | 9,112/923,000 | | | 0.80(0.67,0.94) | | RR | 0.75[0.67 to 0.85] | | 32/6/4/22 | random | 73.0%;0.000 | NA |
| Energy intake | | | IV | highest *vs.* lowest | | 1 | Yu 2012 | 814/61,087 | | | NA | | RR | 1.19[1.08 to 1.31] | | 2/0/2/0 | NA | NA; NA | NA |
| Chili Consumption | | | IV | highest *vs.* lowest | | 6 | Lei 2021 | 3317/8,337 | | | 1.92(1.21,2.64) | | OR | 1.51[1.02 to 2.00] | | 16/0/1/15 | random | 71.9%;0.000 | 0.594 |
| Refrigerator use | | | IV | use vs. non-use | | 1 | Yan 2018 | 3,987/14,361 | | | 0.89(0.84,0.95) | | OR | 0.70[0.56 to 0.88] | | 12/0/1/11 | random | 89.8%;0.000 | 0.183 |
| Gastric atrophy | | | III | yes vs. no | | 1 | Islami 2011 | 1061/NA | | | 1.58(0.95,2.63) | | RR | 2.89[2.09 to 3.98] | | 13/0/1/12 | random | 34%;0.11 | 0.65 |
| Ginseng consumption | | | III | any vs. none | | 2 | Ji 2016 | 23,767/58,889 | | | 0.71(0.62,0.80) | | RR | 0.83[0.75 to 0.92] | | 4/1/2/1 | random | 88.0%；0.000 | NA |
| Papillomavirus infection | | | IV | infection vs. non- infection | | 1 | Bae 2021 | 462/1,736 | | | 3.55(1.70,7.43) | | OR | 5.80[3.27 to 10.31] | | 18/0/0/18 | random | 60.0%;0.001 | 0.013 |
| Dietary cholesterol intake | | | III | highest *vs.* lowest | | 1 | Miao 2021 | 16,490/17,793 | | | 1.59(1.35,1.87) | | OR | 1.35[1.13 to 1.62] | | 14/0/0/14 | random | 70.1%;0.000 | 0.83 |
| Dietary polyphenol intake | | | II | highest *vs.* lowest | | 1 | Fagundes 2022 | 11,610/1,197,857 | | | 0.76(0.65,0.89) | | RR | 0.71[0.62 to 0.81] | | 19/0/7/12 | random | 60.5%;0.000 | 0.316 |
| Dietary fiber intake | | | III | highest *vs.* lowest | | 1 | Zhang 2013 | 6,590/580,064 | | | 0.90(0.70,1.30) | | OR | 0.58[0.49 to 0.67] | | 21/0/2/19 | random | 62.2%;0.000 | 0.931 |
| Education attainment | | | IV | highest vs. lowest | | 2 | Uthman 2013 | NA | | | 1.25(1.13,1.38) | | RII | 2.97[1.93 to 4.58] | | 26/0/9/17 | random | 98.7%;0.000 | NA |
| Combined socioeconomic position | | | IV | high vs. low | | 1 | Uthman 2013 | NA | | | 1.49(1.15,1.93) | | RII | 2.64[1.05 to 6.63] | | 4/0/2/2 | random | 66.4%;0.030 | NA |
| Combined lifestyle factors | | | II | healthiest vs. least healthy lifestyles | | 1 | Zhang 2020 | 2,974/1,178,777 | | | 0.62(0.49,0.79) | | HR | 0.60[0.48 to 0.74] | | 5/0/5/0 | random | 62.1%;0.022 | NA |
| Toothbrushing frequency | | | III | high vs. low | | 1 | Wu 2021 | 16,962/217,811 | | | 0.86(0.78,0.94) | | OR | 0.84[0.77 to 0.92] | | 3/0/2/1 | fixed | 30.8%;0.236 | NA |
| Statins | | | IV | use vs. non-use | | 10 | Chen 2023 | NA | | | 0.70(0.65,0.74) | | OR/RR | 0.74[0.67 to 0.80] | | 23/2/9/12 | random | 85.4%;0.000 | 0.154 |
| Haemoglobin A1c levels | | | IV | HbA1c levels >6% vs.  <6% | | 1 | Zheng 2022 | 821/562,590 | | | 1.00(0.76,1.32) | | HR | 1.36[1.06 to 1.74] | | 5/0/5/0 | random | 43.3%;0.43 | 0.292 |
| Diabetes | | | IV | yes vs. no | | 9 | Guo 2022 | NA | | | NA | | RR | 1.17[1.09 to 1.25] | | 41/0/41/0 | random | 89.1%;＜0.01 | NA |
| T1DM | | | IV | yes vs. no | | 9 | Guo 2022 | NA | | | 1.37(1.01,1.87) | | RR | 1.46[1.22 to1.74] | | 7/0/7/0 | fixed | 23.5%;0.250 | 0.808 |
| T2DM | | | IV | yes vs. no | | 9 | Guo 2022 | NA | | | 1.13(1.10,1.15) | | RR | 1.14[1.06 to 1.22] | | 34/0/34/0 | random | 90.6%;0.000 | 0.808 |
| Cigarette smoking | | | IV | current vs. never | | 11 | Poorolajal 2020 | NA | | | 1.88(1.79,1.97) | | OR | 1.61[1.49 to 1.75] | | 95/0/4/91 | random | 78.0%;0.000 | NA |
| Selenium exposure | | | IV | high vs. low | | 1 | Gong 2016 | NA/7,778 | | | NA | | OR | 0.87[0.78 to 0.97] | | 4/0/4/0 | fixed | 25.0%;0.25 | 0.112 |
| Hormone replacement therapy | | | I | user vs. non-user | | 4 | Jang 2022 | NA/1,919,089 | | | 0.61(0.50,0.74) | | RR | 0.72[0.64 to 0.81] | | 11/0/7/4 | random | 2.21%;0.87 | 0.416 |
| TC | | | I | highest vs. lowest | | 1 | Xu 2023 | 40,656/4,243,457 | | | 0.92(0.88,0.96) | | HR | 0.89[0.87 to 0.92] | | 8/0/8/0 | fixed | 15.0%;0.000 | 0.31 |
| HDL-C | | | I | highest vs. lowest | | 1 | Xu 2023 | 19,196/3,484,919 | | | 0.90(0.87,0.94) | | HR | 0.90[0.86 to 0.93] | | 4/0/4/0 | fixed | 0%;0.67 | 0.18 |
| Tooth loss | | | IV | highest vs. lowest | | 2 | Shi 2018 | 2,174/123,689 | | | NA | | RR | 1.09[1.03 to 1.16] | | 9/0/5/4 | random | 0%;0.763 | 0.89 |
| Pickled food | | | IV | high vs. low | | 12 | Wu 2021 | NA/570,390 | | | NA | | RR | 1.28[1.05 to 1.57] | | 12/0/10/2 | random | 79.4%;0.000 | NA |
| Food-nitrite intake | | | IV | highest vs. lowest | | 15 | Seyyedsalehi 2023 | NA | | | 0.82(0.70,0.96) | | OR | 1.33[1.02 to 1.73] | | 13/0/4/9 | random | 92.2%;0.000 | NA |
| Dietary salt intake | | | III | high vs. low | | 9 | Wu 2022 | NA/37,225 | | | 1.07(0.95,1.20) | | OR | 1.55[1.45 to 1.64] | | 38/0/0/38 | random | 82.8%;0.000 | NA |
| Dietary folate intake | | | III | highest vs. lowest | | 3 | Liu 2017 | 6,881/751,257 | | | 0.62(0.47,0.82) | | OR | 0.76[0.65 to 0.88] | | 21/0/5/16 | random | 67.6%;0.000 | 0.015 |
| Use of insulin | | | IV | Insulin vs. no insulin | | 1 | Karlstad 2013 | NA | | | NA | | RR | 1.65[1.02 to 2.68] | | 3/0/2/1 | Random | NA | NA |
| Green tea consumption | | | III | drinking vs. non-drinking | | 10 | Poorolajal 2020 | NA/59,576 | | | 0.95(0.86,1.06) | | OR | 0.88[0.80 to 0.97] | | 16/0/0/16 | random | 22.0%;0.22 | NA |
| Alcohol consumption | | | IV | any vs. none | | 16 | Deng 2021 | NA | | | 0.96(0.90,1.04) | | OR | 1.27[1.08 to 1.50] | | 81/0/13/68 | random | 69.0%;0.000 | NA |
| Helicobacter pylori infection^d^ | | | II | yes vs. no | | 10 | Han 2023 | 1,371/31017 | | | 2.58(1.72,3.86) | | OR | 2.86[2.26 to 3.63] | | 16/0/2/14 | random | 41.0%;0.04 | NA |
| Helicobacter pylori infection^e^ | | | II | yes vs. no | | 10 | Han 2023 | 12,590/53,838 | | | 4.93(4,34,5.60) | | OR | 4.36[3.54 to 5.37] | | 26/0/4/22 | random | 83%;0.000 | NA |
| Helicobacter pylori infection^f^ | | | II | yes vs. no | | 10 | Han 2023 | 7,759/13,368 | | | 0.57(0.44,0.75) | | OR | 4.03[2.59 to 6.27] | | 24/0/2/22 | random | 92%;0.000 | NA |
| Depression | | | II | depression vs. non- depression | | 1 | Zhang 2022 | 48,592/ 55,482 | | | 1.32(1.19,1.48) | | OR | 1.84[1.61 to 2.09] | | 22/0/2/22 | random | 75.0%;0.000 | 0.177 |
| Proton pump inhibitors | | | III | user vs. non-user | | 12 | Peng 2023 | 1,912,492/2,938,999 | | | 3.38(3.25,3.52) | | OR | 1.75[1.28 to 2.40] | | 16/0/8/8 | random | 97.0%;0.000 | 0.101 |
| Dermatomyositis | | | II | yes vs. no | | 2 | Zadori 2021 | NA/2,130 | | | 3.50(1.70,7.30) | | SIR | 3.71[2.04 to 6.75] | | 4/0/0/4 | random | 14%;0.32 | NA |
| Inflammatory myopathies | | | IV | yes vs. no | | 2 | Zadori 2021 | NA/13,775 | | | 3.50(1.70,7.30) | | SIR | 2.68[1.40 to 5.12] | | 7/0/1/6 | random | 37%;0.14 | NA |
| Systemic lupus erythematosus | | | IV | yes vs. no | | 2 | Zadori 2021 | NA | | | 2.08(1,97,2.19) | | SIR | 1.48[1.09 to 2.01] | | 7/0/3/4 | random | 60%;0.04 | NA |
| Graves’ disease | | | II | yes vs. no | | 2 | Zadori 2021 | NA/73,618 | | | 1.24(1.08,1.42) | | SIR | 1.28[1.16 to 1.41] | | 3/0/2/1 | random | 0%;0.82 | NA |
| ***Non-Significant associations*** | | | | | | | | | | | | | | | | | | | |
| 2,4-Dichlorophenoxyacetic acid exposure | | | NS | exposure vs. non- exposure | | 1 | Goodman 2015 | 274/2,237 | | | 1.85(1.05,3.25) | | RR | 1.14 [0.62 to 2.10] | | 3/0/1/2 | random | 54.9%; 0.109 | NA |
| White meat consumption | | | NS | per 100 g/day increase | | 2 | Kim 2019 | 6,802/1,580,366 | | | 0.87(0.69,1.10) | | RR | 0.86[0.64 to 1.15] | | 12/0/4/8 | random | 52.8%; 0.010 | 0.096 |
| Fish consumption | | | NS | ≥1time/wk vs. <1time/wk | | 5 | Poorolajal 2020 | NA/15,250 | | | 0.78(0.71,0.85) | | OR | 0.79 [0.61 to 1.03] | | 11/0/11/0 | random | 76%; 0.0000 | NA |
| Total vegetable intake | | | NS | 100 g/day increase | | 9 | Naemi 2023 | 7,075/1,445,175 | | | 1.05(0.99,1.12) | | RR | 0.96 [0.92 to 1.00] | | 10/0/10/0 | random | 37.4%; 0.101 | NA |
| Citrus fruit intake | | | NS | 50 g/day increase | | 2 | Naemi 2023 | 2837/1,147,546 | | | 0.97(0.90,1.04) | | RR | 0.98 [0.94 to 1.02] | | 6/0/6/0 | random | 0.0%; 0.583 | NA |
| Dietary flavonoid intake | | | NS | high vs. low | | 8 | Wang 2021 | 4,868/587,078 | | | 0.85(0.69,1.05) | | RR | 0.92 [0.79 to 1.02] | | 8/0/3/5 | random | 50.9%; 0.032 | 0.917 |
| Dairy product consumption | | | NS | highest *vs.* lowest | | 4 | Sun 2014 | 9,053/102,089 | | | 0.95(0.87,1.05) | | OR | 1.06 [0.95 to 1.18] | | 39/0/10/29 | random | 67.1%; 0.000 | 0.135 |
| Milk consumption | | | NS | highest *vs.* lowest | | 4 | Sun 2014 | 8,031/265,602 | | | 1.02(0.16,7.08) | | OR | 1.11[0.94 to 1.31] | | 23/0/7/16 | random | 70.1%;0.000 | NA |
| Cheese consumption | | | NS | highest *vs.* lowest | | 1 | Sun 2014 | 2,913/117,492 | | | 0.92(0.67,1.26) | | OR | 0.95 [0.80 to 1.12] | | 9/0/2/7 | random | 9.1%;0.360 | 0.621 |
| MetS | | | NS | yes *vs.* no | | 2 | Mariani 2020 | 4,895,736/88,712,302 | | | 1.26(1.20,1.32) | | RR | 1.05 [0.92 to 1.18] | | 7/0/7/0 | random | 74.2%; 0.000 | NA |
| Lycopene | | | NS | highest vs. lowest | | 2 | Zhou 2016 | 966/23,528 | | | 1.02(0.66,1.58) | | RR | 0.80[0.60 to 1.07] | | 4/0/4/0 | fixed | 0.0%;0.446 | NA |
| BMI | | | NS | ≥25.0kg/m^2^ vs. 18.5-24.9 kg/m^2^ | | 6 | Poorolajal 2020 | NA/147,410 | | | 1.30(1.12,1.52) | | OR | 0.89[0.74 to 1.08] | | 14/0/2/12 | random | 86.0%;0.000 | NA |
| Body under-weight^c^ | | | NS | underweight vs. normal weight | | 1 | Azizi 2023 | NA/13,804,314 | | | 1.17(1.10,1.24) | | RR | 1.12[0.97 to 1.30] | | 11/0/11/0 | random | 89.08%; NA | NA |
| Anthocyanins intake | | | NS | highest *vs.* lowest | | 1 | Yang 2019 | 3,243/951,216 | | | 0.94(0.72,1.23) | | RR | 0.92[0.81 to 1.04] | | 6/0/2/4 | random | 0.0%;0.597 | <0.10 |
| Nut consumption | | | NS | per 10 g/d increment | | 1 | Cao 2023 | 2,184/490,455 | | | NA | | OR | 0.98[0.96 to 1.01] | | 4/0/2/2 | random | NA | NA |
| Coffee consumption | | | NS | per 1 cup/d increment | | 11 | Song 2022 | 3,266/1,520,562 | | | 1.00(0.99,1.02) | | RR | 1.00[0.99 to 1.01] | | 14/0/14/0 | random | 31.6%;0.123 | NA |
| Vitamin B-12 | | | NS | highest *vs.* lowest | | 2 | He 2022 | 3,494/611,638 | | | 1.33(1.15,1.55) | | OR | 0.88[0.69 to 1.12] | | 9/0/3/6 | random | 74.9%;0.000 | 0.551 |
| Vitamin D intake | | | NS | ≥20 ng/mL vs. <12 ng/mL | | 4 | Chen 2022 | 512/59,293 | | | 1.11(0.80,1.55) | | OR | 0.93[0.77 to 1.11] | | 5/0/3/2 | random | 25.7%;0.192 | 0.005 |
| Hexavalent chromium exposure | | | NS | exposure vs. non- exposure | | 3 | Suh 2019 | NA/144,611 | | | 1.34(1.12,1.61) | | RR | 1.08[0.96 to 1.21] | | 44/0/41/3 | random | 25.1%; NA | 0.57 |
| Dietary meat mutagens intake | | | NS | high vs. low | | 1 | Reng 2022 | 1,252/338,893 | | | NR | | OR | 1.68[0.92 to 3.08] | | 4/0/1/3 | random | 80.6%;0.000 | NA |
| Dietary fat intake | | | NS | highest *vs.* lowest | | 1 | Han 2015 | 8,128/514,752 | | | 1.33(1.12,1.57) | | RR | 1.18[1.00 to 1.39] | | 21/0/1/20 | random | 69.5%;0.000 | 0.43 |
| Income levels | | | NS | high vs. low | | 1 | Uthman 2013 | NA | | | 1.00(0.99,1.00) | | RII | 1.25[0.93 to 1.68] | | 10/0/2/8 | random | 83.3%;0.000 | NA |
| Asbestos exposure | | | NS | any vs. none | | 1 | Fortunato 2015 | NA/73,361 | | | NA | | SIR | 1.07[0.91 to 1.25] | | 15/0/15/0 | random | 25.5%;0.26 | NA |
| Bisphosphonates | | | NS | any vs. none | | 3 | Cai 2017 | 109,258/459,200 | | | 0.98(0.76,1.27) | | OR | 1.00[0.86 to 1.15] | | 9/0/4/5 | random | 54.2%;0.010 | 0.888 |
| Carbohydrate intake | | | NS | highest vs. lowest | | 1 | Ye 2017 | 6,675/80,094 | | | 1.65(1.28,2.12) | | RR | 1.17[0.91 to 1.50] | | 22/0/2/20 | random | 79.1%;0.000 | 0.784 |
| Serum glucose levels | | | NS | highest vs. lowest | | 1 | Zheng 2022 | 4787/3,075,546 | | | 1.00(.91,1.10) | | HR | 1.11[0.98 to 1.26] | | 7/0/6/1 | random | 70.1%;0.001 | 0.055 |
| Rubber exposure | | | NS | ever vs. none | | 1 | Boniol 2017 | NA | | | NA | | RR | 1.06[0.82 to 1.36] | | 10/0/8/2 | random | 8%; NA | NA |
| Zinc intake | | | NS | highest vs. lowest | | 1 | Li 2014 | 1,573/2,519 | | | 1.25(1.02,1.53) | | RR | 0.91[0.64 to1.29] | | 7/0/0/7 | random | 77.6%;0.000 | NA |
| Glycemic Index | | | NS | highest vs. lowest | | 2 | Turati 2019 | NA/3,152 | | | 0.90(0.74,1.11) | | RR | 1.09[0.79 to 1.52] | | 7/0/0/7 | random | 80.3%;0.000 | NA |
| Glycemic load | | | NS | highest vs. lowest | | 2 | Turati 2019 | NA/3,152 | | | 1.06(0.82,1.38) | | RR | 1.04[0.80 to 1.35] | | 7/0/0/7 | random | 48.1%;0.061 | NA |
| TGs | | | NS | highest vs. lowest | | 1 | Xu 2023 | 19,177/3,611,979 | | | 0.99(0.95,1.04) | | HR | 1.00[0.96 to 1.04] | | 4/0/4/0 | fixed | 37.0%;0.19 | 0.12 |
| LDL-C | | | NS | highest vs. lowest | | 1 | Xu 2023 | 18,573/3,331,682 | | | 0.96(0.92,1.01) | | HR | 0.96[0.91 to 1.00] | | 3/0/3/0 | fixed | 0%;0.47 | 0.31 |
| Food-NDMA intake | | | NS | highest vs. lowest | | 15 | Seyyedsalehi 2023 | 2,014/410,682 | | | 1.02(0.86,1.21) | | OR | 1.37[0.87 to 2.16] | | 8/0/3/5 | random | 94.4%;0.000 | NA |
| Food-nitrate intake | | | NS | highest vs. lowest | | 15 | Seyyedsalehi 2023 | 3,092/699,305 | | | 0.87(0.73,1.04) | | OR | 1.13[0.81 to 1.57] | | 12/0/4/8 | random | 93.2%;0.000 | NA |
| Water-nitrate intake | | | NS | highest vs. lowest | | 15 | Seyyedsalehi 2023 | 435/2,147 | | | 1.12(0.83,1.52) | | OR | 0.60[0.18 to 1.94] | | 3/0/1/2 | random | 93.5%;0.000 | NA |
| Inflammatory bowel disease | | | NS | yes vs. no | | 1 | Wan 2021 | NA | | | NA | | OR | 0.87[0.62 to 1.13] | | 7/0/6/1 | Random | 0%; NA | 0.13 |
| Black tea consumption | | | NS | drinking vs. non-drinking | | 10 | Poorolajal 2020 | NA/40,079 | | | 0.95(0.81,1.12) | | OR | 1.00[0.84 to 1.20] | | 15/0/0/15 | random | 62.0%;0.002 | NA |
| Parity | | | NS | ever parity *vs.* nulliparous | | 1 | Chen 2016 | 4928/2,500,465 | | | 1.01(0.89,1.05) | | RR | 0.96[0.87 to 1.05] | | 7/0/7/0 | random | 0%;0.504 | 0.159 |
| Bitumen exposure | | | NS | yes vs. no | | 2 | Mundt 2018 | NA | | | 0.97(0.82,1.15) | | RR | 1.14[0.99 to 1,31] | | 21/0/15/6 | random | 44.8%;0.015 | 0.030 |
| Cement exposure | | | NS | yes vs. no | | 1 | Cohen 2014 | NA/6,768 | | | 1.70(0.97,2.76) | | SIR | 1.05[0.66 to 1.68] | | 4/0/4/0 | random | 46.2%;0.134 | 0.03 |
| Abbreviations | EB, Epstein-Barr; RR, relative risk; OR, odds ratio; HR, hazard ratios; SIR, standardized incidence rate; RII, relative indexes of inequality; NA, not available; NSAIDs, non-steroidal anti-inflammatory drugs; IIM, incomplete intestinal metaplasia; CIM, complete intestinal metaplasia; MAFLD, metabolic-associated fatty liver disease; MDS, mediterranean diet score; DII, dietary inflammatory index; T1DM, Type 1 diabetes mellitus; T2DM, Type 2 diabetes mellitus;  TC, total cholesterol; HDL-C, high-density lipoprotein cholesterol; MetS, metabolic syndrome; BMI, body mass index; TGs, triglycerides; NDMA, N-nitrosodimethylamine; LDL-C, low-density lipoprotein cholesterol. | | | | | | | | | | | | | | | | | | |
| Note | ^^^ Number of studies.  ^#^ 95% confidence interval of largest study in each meta-analysis.  ^φ^ Summary risk ratio (95% confidence interval).  ^§^ T, total No. of studies; R, randomized controlled trial; C, cohort studies; P, population/hospital-based case-control and/or cross-sectional.  ^a^ WHO category (≥30)/ Asian-Pacific category (≥25) kg/m^2^  ^b^ WHO category (25-29.9)/ Asian-Pacific category (23-24.9) kg/m^2^  ^c^ BMI<18.5 kg/m^2^  ^d^ CGC in East Asia  ^e^ NCGC in East Asia  ^f^ NCGC in West | | | | | | | | | | | | | | | | | | |
