# Supplementary material for: Risk factors for gastric cancer: an umbrella review of systematic reviews and meta-analyses
Source: Front Oncol. 2025 Jun 26;15:1564575. doi: 10.3389/fonc.2025.1564575 (PMC12241803; doi:10.3389/fonc.2025.1564575)
Supplement: Supplementary file 2 [file Table2.docx]

| Supplementary Table S2. Assessments of AMSTAR scores. | | | | | | | | | | | | | | | | | | |
| --- | --- | --- | --- | --- | --- | --- | --- | --- | --- | --- | --- | --- | --- | --- | --- | --- | --- | --- |
| Risk Factors | Outcome types | Assessed with | Author | Year | A priori design provided | Duplicate study selection & data extraction | At least two electronic databases searched | Status of  publication used as an inclusion criterion | List of  included and excluded studies provided | | Characteristics of included  studies provided | Scientific quality of  included studies assessed | Scientific quality of the included studies used  appropriately to form  conclusions | Appropriate methods to  combine studies | Publication bias assessed | Conflict of interest included | | Total AMSTAR Score |
| 2,4-Dichlorophenoxyacetic acid exposure | GC | exposure vs. non-exposure | Goodman | 2015 | 0 | 1 | 1 | 0 | 1 | 1 | | 1 | 0 | 1 | 1 | | 1 | 8 |
| EB virus | GC | infection vs. non-infection | Tavakoli | 2020 | 0 | 1 | 1 | 0 | 1 | 1 | | 1 | 0 | 1 | 1 | | 1 | 8 |
| NSAIDs | cardia and non-cardia GC | use vs. non-use | Huang | 2017 | 0 | 1 | 1 | 0 | 1 | 1 | | 1 | 0 | 1 | 1 | | 1 | 8 |
| Aspirin | GC | use vs. non-use | Niikura | 2020 | 0 | 1 | 1 | 0 | 1 | 1 | | 1 | 0 | 1 | 1 | | 1 | 8 |
| Non-aspirin NSAIDs | cardia and non-cardia GC | use vs. non-use | Huang | 2017 | 0 | 1 | 1 | 0 | 1 | 1 | | 1 | 0 | 1 | 1 | | 1 | 8 |
| Red meat consumption | cardia and non-cardia GC | per 100 g/day increase | Kim | 2019 | 0 | 1 | 1 | 0 | 1 | 1 | | 1 | 0 | 1 | 1 | | 1 | 8 |
| Processed meat consumption | cardia and non-cardia GC | per 50 g/day increase | Kim | 2019 | 0 | 1 | 1 | 0 | 1 | 1 | | 1 | 0 | 1 | 1 | | 1 | 8 |
| White meat consumption | cardia and non-cardia GC | per 100 g/day increase | Kim | 2019 | 0 | 1 | 1 | 0 | 1 | 1 | | 1 | 0 | 1 | 1 | | 1 | 8 |
| Intestinal metaplasia | GC | IIM vs. CIM | Du | 2021 | 1 | 1 | 1 | 0 | 1 | 1 | | 1 | 0 | 1 | 1 | | 1 | 9 |
| Fish consumption | GC | ≥1time/wk vs. <1time/wk | Poorolajal | 2020 | 0 | 1 | 1 | 0 | 1 | 1 | | 1 | 0 | 1 | 1 | | 1 | 8 |
| Total vegetable intake | cardia and non-cardia GC | 100 g/day increase | Naemi | 2023 | 0 | 1 | 1 | 0 | 1 | 1 | | 1 | 0 | 1 | 1 | | 1 | 8 |
| Fruit intake | cardia and non-cardia GC | 100 g/day increase | Naemi | 2023 | 0 | 1 | 1 | 0 | 1 | 1 | | 1 | 0 | 1 | 1 | | 1 | 8 |
| Vegetable and fruit intake | cardia and non-cardia GC | 200 g/day increase | Naemi | 2023 | 0 | 1 | 1 | 0 | 1 | 1 | | 1 | 0 | 1 | 1 | | 1 | 8 |
| Citrus fruit intake | cardia and non-cardia GC | 50 g/day increase | Naemi | 2023 | 0 | 1 | 1 | 0 | 1 | 1 | | 1 | 0 | 1 | 1 | | 1 | 8 |
| Allium vegetable intake | cardia and non-cardia GC | highest vs. lowest | Turati | 2015 | 0 | 1 | 1 | 0 | 1 | 1 | | 1 | 0 | 1 | 1 | | 1 | 8 |
| Cruciferous vegetable | GC | highest vs. lowest | Wu | 2013 | 0 | 1 | 1 | 0 | 1 | 1 | | 1 | 0 | 1 | 1 | | 1 | 8 |
| Soy food intake | GC | high vs. low | Wang | 2021 | 1 | 1 | 1 | 0 | 1 | 1 | | 1 | 0 | 1 | 1 | | 0 | 8 |
| Fermented soy food | GC | high vs. low | Wang | 2021 | 1 | 1 | 1 | 0 | 1 | 1 | | 1 | 0 | 1 | 1 | | 0 | 8 |
| Non-fermented soy food | GC | high vs. low | Wang | 2021 | 1 | 1 | 1 | 0 | 1 | 1 | | 1 | 0 | 1 | 1 | | 0 | 8 |
| Dietary flavonoid intake | GC | high vs. low | Wang | 2021 | 1 | 1 | 1 | 0 | 1 | 1 | | 1 | 0 | 1 | 1 | | 0 | 8 |
| Dairy product consumption | cardia and non-cardia GC | highest *vs.* lowest | Sun | 2014 | 0 | 0 | 1 | 0 | 1 | 1 | | 1 | 0 | 1 | 1 | | 0 | 6 |
| Milk consumption | cardia and non-cardia GC | highest *vs.* lowest | Sun | 2014 | 0 | 0 | 1 | 0 | 1 | 1 | | 1 | 0 | 1 | 1 | | 0 | 6 |
| Cheese consumption | cardia and non-cardia GC | highest *vs.* lowest | Sun | 2014 | 0 | 0 | 1 | 0 | 1 | 1 | | 1 | 0 | 1 | 1 | | 0 | 6 |
| Garlic consumption | GC | Any vs. none | Huang | 2022 | 1 | 1 | 1 | 0 | 1 | 1 | | 1 | 0 | 1 | 1 | | 1 | 9 |
| MAFLD | GC | yes *vs.* no | Zou | 2023 | 1 | 1 | 1 | 0 | 1 | 1 | | 1 | 0 | 1 | 1 | | 1 | 9 |
| MetS | GC | yes *vs.* no | Mariani | 2020 | 1 | 1 | 1 | 0 | 1 | 1 | | 1 | 0 | 1 | 1 | | 1 | 9 |
| Cholecystectomy | cardia and non-cardia GC | yes *vs.* no | Yang | 2022 | 0 | 1 | 1 | 0 | 1 | 1 | | 1 | 0 | 1 | 1 | | 1 | 8 |
| Healthy dietary pattern | GC | highest *vs.* lowest | Shu | 2013 | 0 | 1 | 1 | 0 | 1 | 1 | | 1 | 0 | 1 | 1 | | 0 | 7 |
| Western-style dietary pattern | GC | highest *vs.* lowest | Shu | 2013 | 0 | 1 | 1 | 0 | 1 | 1 | | 1 | 0 | 1 | 1 | | 0 | 7 |
| Alcohol-drinking dietary pattern | GC | highest *vs.* lowest | Shu | 2013 | 0 | 1 | 1 | 0 | 1 | 1 | | 1 | 0 | 1 | 1 | | 0 | 7 |
| MDS | GC | highest *vs.* lowest | Moazzen | 2020 | 0 | 1 | 1 | 0 | 1 | 1 | | 1 | 0 | 1 | 1 | | 1 | 8 |
| DII | GC | highest *vs.* lowest | Moazzen | 2020 | 0 | 1 | 1 | 0 | 1 | 1 | | 1 | 0 | 1 | 1 | | 1 | 8 |
| Physical activity | GC | high vs. low | Xie | 2020 | 1 | 1 | 1 | 0 | 1 | 1 | | 1 | 0 | 1 | 1 | | 1 | 9 |
| Pernicious anaemia | GC | yes vs. no | Vannella | 2013 | 0 | 1 | 0 | 0 | 1 | 1 | | 1 | 0 | 1 | 1 | | 1 | 7 |
| Metformin | GC | use vs. non-use | Seo | 2022 | 0 | 1 | 1 | 0 | 1 | 1 | | 1 | 0 | 1 | 1 | | 1 | 8 |
| Tomato products consumption | cardia and non-cardia GC | high vs. low | Yang | 2013 | 0 | 1 | 1 | 0 | 0 | 1 | | 1 | 0 | 1 | 1 | | 0 | 6 |
| Lycopene | GC | highest *vs.* lowest | Zhou | 2016 | 0 | 1 | 1 | 0 | 1 | 1 | | 1 | 0 | 1 | 1 | | 1 | 8 |
| BMI | GC | overweight/obesity vs. normal weight | Poorolajal | 2020 | 0 | 1 | 1 | 0 | 1 | 1 | | 1 | 0 | 1 | 1 | | 1 | 8 |
| Body obesity | cardia and non-cardia GC | obesity vs. normal weight | Azizi | 2023 | 1 | 1 | 1 | 1 | 1 | 1 | | 1 | 0 | 1 | 1 | | 1 | 10 |
| Body over-weight | cardia and non-cardia GC | overweight vs. normal weight | Azizi | 2023 | 1 | 1 | 1 | 1 | 1 | 1 | | 1 | 0 | 1 | 1 | | 1 | 10 |
| Body under-weight | cardia and non-cardia GC | underweight vs. normal weight | Azizi | 2023 | 1 | 1 | 1 | 1 | 1 | 1 | | 1 | 0 | 1 | 1 | | 1 | 10 |
| Whole grains consumption | GC | intake vs. non-intake | Wang | 2020 | 0 | 1 | 1 | 0 | 1 | 1 | | 1 | 0 | 1 | 1 | | 1 | 8 |
| Refined grains consumption | GC | ≥3times/wk vs.＜3times/wk | Wang | 2020 | 0 | 1 | 1 | 0 | 1 | 1 | | 1 | 0 | 1 | 1 | | 1 | 8 |
| Anthocyanins intake | cardia and non-cardia GC | highest vs. lowest | Yang | 2019 | 0 | 1 | 1 | 0 | 1 | 1 | | 1 | 1 | 1 | 1 | | 0 | 8 |
| Talc exposure | GC | any vs. none | Chang | 2020 | 0 | 1 | 1 | 0 | 1 | 1 | | 1 | 0 | 1 | 1 | | 1 | 8 |
| Nut consumption | cardia and non-cardia GC | highest *vs.* lowest | Cao | 2023 | 0 | 1 | 1 | 0 | 1 | 1 | | 1 | 0 | 1 | 1 | | 1 | 8 |
| Crystalline silica exposure | GC | exposure vs. non- exposure | Lee | 2016 | 0 | 1 | 1 | 0 | 1 | 1 | | 1 | 0 | 1 | 1 | | 0 | 7 |
| Coffee consumption | GC | per 1 cup/d increment | Song | 2022 | 0 | 1 | 1 | 0 | 1 | 1 | | 1 | 0 | 1 | 1 | | 1 | 8 |
| β-carotene intake | GC | highest *vs.* lowest | Zhou | 2016 | 0 | 1 | 1 | 0 | 1 | 1 | | 1 | 0 | 1 | 1 | | 1 | 8 |
| Vitamin intake | GC | highest *vs.* lowest | Kong | 2014 | 0 | 1 | 1 | 0 | 1 | 1 | | 1 | 0 | 1 | 1 | | 1 | 8 |
| Vitamin A intake | GC | highest *vs.* lowest | Wu | 2015 | 0 | 1 | 1 | 0 | 1 | 1 | | 1 | 0 | 1 | 1 | | 1 | 8 |
| Vitamin B-12 | GC | highest *vs.* lowest | He | 2022 | 0 | 1 | 1 | 0 | 1 | 1 | | 1 | 0 | 1 | 1 | | 1 | 8 |
| Vitamin C intake | GC | highest *vs.* lowest | Kong | 2014 | 0 | 1 | 1 | 0 | 1 | 1 | | 1 | 0 | 1 | 1 | | 1 | 8 |
| Vitamin D intake | GC | ≥20 ng/mL vs. <12 ng/mL | Chen | 2022 | 1 | 1 | 1 | 0 | 1 | 1 | | 1 | 0 | 1 | 1 | | 1 | 9 |
| Vitamin E intake | GC | highest *vs.* lowest | Kong | 2014 | 0 | 1 | 1 | 0 | 1 | 1 | | 1 | 0 | 1 | 1 | | 1 | 8 |
| Energy intake | GC | highest *vs.* lowest | Yu | 2012 | 0 | 1 | 1 | 0 | 1 | 1 | | 1 | 0 | 1 | 1 | | 0 | 8 |
| Chili consumption | GC | highest *vs.* lowest | Lei | 2021 | 0 | 1 | 1 | 1 | 1 | 1 | | 0 | 1 | 1 | 1 | | 1 | 9 |
| Refrigerator use | GC | use vs. non-use | Yan | 2018 | 0 | 1 | 1 | 0 | 1 | 1 | | 1 | 0 | 1 | 1 | | 0 | 7 |
| Gastric atrophy | GC | yes vs. no | Islami | 2011 | 0 | 1 | 1 | 0 | 1 | 1 | | 1 | 0 | 1 | 1 | | 1 | 8 |
| Hexavalent chromium exposure | GC | exposure vs. non- exposure | Suh | 2019 | 1 | 1 | 1 | 1 | 1 | 1 | | 1 | 0 | 1 | 1 | | 1 | 10 |
| Ginseng consumption | GC | Any vs. none | Ji | 2016 | 0 | 1 | 1 | 1 | 1 | 1 | | 1 | 0 | 1 | 1 | | 1 | 9 |
| Dietary meat mutagens intake | GC | high vs. low | Reng | 2022 | 1 | 1 | 1 | 0 | 1 | 1 | | 1 | 0 | 1 | 1 | | 1 | 9 |
| Papillomavirus infection | GC | infection vs. non- infection | Bae | 2021 | 0 | 1 | 0 | 0 | 0 | 1 | | 1 | 0 | 1 | 1 | | 1 | 6 |
| Dietary cholesterol intake | GC | highest *vs.* lowest | Miao | 2021 | 1 | 1 | 1 | 1 | 1 | 1 | | 1 | 0 | 1 | 1 | | 1 | 10 |
| Dietary polyphenol intake | GC | highest *vs.* lowest | Fagundes | 2022 | 1 | 1 | 1 | 0 | 1 | 1 | | 1 | 0 | 1 | 1 | | 1 | 9 |
| Dietary fiber intake | GC | highest *vs.* lowest | Zhang | 2013 | 0 | 1 | 1 | 0 | 1 | 1 | | 1 | 0 | 1 | 1 | | 1 | 8 |
| Dietary fat intake | GC | highest *vs.* lowest | Han | 2015 | 0 | 1 | 1 | 0 | 1 | 1 | | 1 | 0 | 1 | 1 | | 0 | 7 |
| Education attainment | GC | highest vs. lowest | Uthman | 2013 | 0 | 1 | 1 | 0 | 1 | 1 | | 1 | 0 | 1 | 1 | | 1 | 8 |
| Income levels | GC | highest vs. lowest | Uthman | 2013 | 0 | 1 | 1 | 0 | 1 | 1 | | 1 | 0 | 1 | 1 | | 1 | 8 |
| Combined socioeconomic position | GC | highest vs. lowest | Uthman | 2013 | 0 | 1 | 1 | 0 | 1 | 1 | | 1 | 0 | 1 | 1 | | 1 | 8 |
| Combined lifestyle factors | GC | healthiest vs. least healthy lifestyles | Zhang | 2020 | 0 | 1 | 1 | 0 | 1 | 1 | | 1 | 0 | 1 | 1 | | 1 | 8 |
| Asbestos exposure | GC | any vs. none | Fortunato | 2015 | 0 | 0 | 1 | 0 | 0 | 1 | | 1 | 0 | 1 | 1 | | 1 | 6 |
| Toothbrushing frequency | GC | high vs. low | Wu | 2021 | 0 | 1 | 1 | 0 | 1 | 1 | | 1 | 0 | 1 | 1 | | 1 | 8 |
| Bisphosphonates | GC | any vs. none | Cai | 2017 | 0 | 1 | 1 | 0 | 1 | 1 | | 0 | 0 | 1 | 1 | | 1 | 7 |
| Statins | GC | use vs. non-use | Chen | 2023 | 0 | 1 | 1 | 0 | 1 | 1 | | 1 | 0 | 1 | 1 | | 1 | 8 |
| Carbohydrate intake | GC | highest vs. lowest | Ye, Yao | 2017 | 0 | 1 | 1 | 0 | 1 | 1 | | 1 | 0 | 1 | 1 | | 1 | 8 |
| Haemoglobin A1c levels | GC | HbA1c levels >6% vs.  <6% | Zheng | 2022 | 1 | 1 | 1 | 1 | 1 | 1 | | 1 | 0 | 1 | 1 | | 1 | 9 |
| Serum glucose levels | GC | highest vs. lowest | Zheng | 2022 | 1 | 1 | 1 | 1 | 1 | 1 | | 1 | 0 | 1 | 1 | | 1 | 9 |
| Diabetes | GC | yes vs. no | Guo | 2022 | 1 | 1 | 1 | 0 | 1 | 1 | | 1 | 0 | 1 | 1 | | 1 | 8 |
| T1DM | GC | yes vs. no | Guo | 2022 | 1 | 1 | 1 | 0 | 1 | 1 | | 1 | 0 | 1 | 1 | | 1 | 8 |
| T2DM | GC | yes vs. no | Guo | 2022 | 1 | 1 | 1 | 0 | 1 | 1 | | 1 | 0 | 1 | 1 | | 1 | 8 |
| Cigarette smoking | GC | current vs. never | Poorolaja | 2020 | 0 | 1 | 1 | 0 | 1 | 1 | | 1 | 0 | 1 | 1 | | 1 | 8 |
| Selenium exposure | cardia and non-cardia GC | high vs. low | Gong | 2016 | 0 | 1 | 1 | 1 | 1 | 1 | | 0 | 0 | 1 | 1 | | 1 | 8 |
| Rubber exposure | GC | ever vs. none | Boniol | 2017 | 0 | 1 | 0 | 0 | 1 | 1 | | 1 | 0 | 1 | 1 | | 1 | 7 |
| Zinc intake | cardia and non-cardia GC | highest vs. lowest | Li | 2014 | 0 | 1 | 1 | 0 | 1 | 1 | | 1 | 0 | 1 | 1 | | 1 | 8 |
| Hormone replacement therapy | GC | user vs. non-user | Jang | 2022 | 1 | 1 | 1 | 0 | 1 | 1 | | 1 | 0 | 1 | 1 | | 1 | 9 |
| Glycemic Index | GC | highest vs. lowest | Turati | 2019 | 0 | 1 | 1 | 0 | 0 | 1 | | 1 | 0 | 1 | 1 | | 1 | 7 |
| Glycemic load | GC | highest vs. lowest | Turati | 2019 | 0 | 1 | 1 | 0 | 0 | 1 | | 1 | 0 | 1 | 1 | | 1 | 7 |
| TGs | GC | highest vs. lowest | Xu | 2023 | 0 | 1 | 1 | 0 | 1 | 1 | | 1 | 0 | 1 | 1 | | 0 | 7 |
| TC | GC | highest vs. lowest | Xu | 2023 | 0 | 1 | 1 | 0 | 1 | 1 | | 1 | 0 | 1 | 1 | | 0 | 7 |
| HDL-C | GC | highest vs. lowest | Xu | 2023 | 0 | 1 | 1 | 0 | 1 | 1 | | 1 | 0 | 1 | 1 | | 0 | 7 |
| LDL-C | GC | highest vs. lowest | Xu | 2023 | 0 | 1 | 1 | 0 | 1 | 1 | | 1 | 0 | 1 | 1 | | 0 | 7 |
| Tooth loss | GC | highest vs. lowest | Shi | 2018 | 0 | 1 | 1 | 0 | 1 | 1 | | 1 | 0 | 1 | 1 | | 1 | 8 |
| Pickled food | GC | high vs. low | Wu | 2021 |  |  |  |  |  |  | |  |  |  |  | |  |  |
| Food-nitrite intake | GC | highest vs. lowest | Seyyedsalehi | 2023 | 0 | 1 | 1 | 0 | 1 | 1 | | 1 | 0 | 1 | 1 | | 1 | 8 |
| Food-nitrate intake | GC | highest vs. lowest | Seyyedsalehi | 2023 | 0 | 1 | 1 | 0 | 1 | 1 | | 1 | 0 | 1 | 1 | | 1 | 8 |
| Food-NDMA intake | GC | highest vs. lowest | Seyyedsalehi | 2023 | 0 | 1 | 1 | 0 | 1 | 1 | | 1 | 0 | 1 | 1 | | 1 | 8 |
| Water-nitrate intake | GC | highest vs. lowest | Seyyedsalehi | 2023 | 0 | 1 | 1 | 0 | 1 | 1 | | 1 | 0 | 1 | 1 | | 1 | 8 |
| Inflammatory bowel disease | GC | yes vs. no | Wan | 2021 | 1 | 1 | 1 | 0 | 1 | 1 | | 1 | 0 | 1 | 1 | | 1 | 9 |
| Dietary salt intake | GC | high vs. low | Wu | 2022 | 0 | 1 | 1 | 0 | 1 | 1 | | 1 | 0 | 1 | 1 | | 1 | 8 |
| Dietary folate intake | cardia and non-cardia GC | highest vs. lowest | Liu | 2017 | 0 | 1 | 1 | 0 | 1 | 1 | | 1 | 1 | 1 | 1 | | 1 | 8 |
| Use of insulin | GC | Insulin vs. no insulin | Karlstad | 2013 | 1 | 1 | 1 | 1 | 1 | 1 | | 1 | 0 | 1 | 1 | | 1 | 10 |
| Green tea consumption | GC | drinking vs. non-drinking | Poorolajal | 2020 | 0 | 1 | 1 | 0 | 1 | 1 | | 1 | 0 | 1 | 1 | | 1 | 8 |
| Black tea consumption | GC | drinking vs. non-drinking | Poorolajal | 2020 | 0 | 1 | 1 | 0 | 1 | 1 | | 1 | 0 | 1 | 1 | | 1 | 8 |
| Alcohol consumption | cardia and non-cardia GC | any vs. none | Deng | 2021 | 0 | 1 | 1 | 0 | 1 | 1 | | 1 | 0 | 1 | 1 | | 1 | 8 |
| Helicobacter pylori infection | cardia and non-cardia GC | yes vs. no | Han | 2023 | 1 | 1 | 1 | 1 | 1 | 1 | | 1 | 0 | 1 | 1 | | 1 | 10 |
| Depression | GC | depression vs. non- depression | Zhang | 2022 | 1 | 1 | 1 | 0 | 1 | 1 | | 1 | 0 | 1 | 1 | | 1 | 9 |
| Proton pump inhibitors | GC | user vs. non-user | Peng | 2023 | 0 | 1 | 1 | 0 | 1 | 1 | | 1 | 0 | 1 | 1 | | 1 | 8 |
| Dermatomyositis | GC | yes vs. no | Zadori | 2021 | 1 | 1 | 1 | 0 | 1 | 1 | | 1 | 0 | 1 | 1 | | 1 | 9 |
| Systemic lupus erythematosus | GC | yes vs. no | Zadori | 2021 | 1 | 1 | 1 | 0 | 1 | 1 | | 1 | 0 | 1 | 1 | | 1 | 9 |
| Inflammatory myopathies | GC | yes vs. no | Zadori | 2021 | 1 | 1 | 1 | 0 | 1 | 1 | | 1 | 0 | 1 | 1 | | 1 | 9 |
| Graves’ disease | GC | yes vs. no | Zadori | 2021 | 1 | 1 | 1 | 0 | 1 | 1 | | 1 | 0 | 1 | 1 | | 1 | 9 |
| Parity | GC | ever parity *vs.* nulliparous | Chen | 2016 | 0 | 1 | 1 | 0 | 1 | 1 | | 1 | 0 | 1 | 1 | | 1 | 8 |
| Bitumen exposure | GC | yes vs. no | Mundt | 2018 | 0 | 0 | 0 | 0 | 1 | 1 | | 1 | 0 | 1 | 1 | | 1 | 6 |
| Cement exposure | GC | yes vs. no | Cohen | 2014 | 0 | 1 | 0 | 0 | 0 | 1 | | 1 | 0 | 1 | 1 | | 1 | 6 |
| Note: GC, gastric cancer; EB, Epstein-Barr; NSAIDs, non-steroidal anti-inflammatory drugs; MAFLD, metabolic-associated fatty liver disease; MetS, metabolic syndrome; MDS, mediterranean diet score; DII, dietary inflammatory index; BMI, body mass index; T1DM, Type 1 diabetes mellitus; T2DM, Type 2 diabetes mellitus; TGs, triglycerides; TC, total cholesterol; HDL-C, high-density lipoprotein cholesterol; LDL-C, low-density lipoprotein cholesterol; NDMA, N-nitrosodimethylamine. | | | | | | | | | | | | | | | | | | |
